# A Comprehensive conceptual and computational dynamics framework for autonomous regeneration of form and function in biological organisms

**DOI:** 10.1093/pnasnexus/pgac308

**Published:** 2023-01-09

**Authors:** Sandhya Samarasinghe, Tran Nguyen Minh-Thai

**Affiliations:** Complex Systems, Big Data and Informatics Initiative (CSBII), Lincoln University, Lincoln 7647, New Zealand; Precision Agriculture Team, Lincoln Agritech Limited, PO Box 69133, Lincoln, New Zealand; Department of Information Systems, College of Information and Communication Technology, Can Tho University, 3/2 Street, Ninh Kieu District, Can Tho, Vietnam

## Abstract

In biology, regeneration is a mysterious phenomenon that has inspired self-repairing systems, robots, and biobots. It is a collective computational process whereby cells communicate to achieve an anatomical set point and restore original function in regenerated tissue or the whole organism. Despite decades of research, the mechanisms involved in this process are still poorly understood. Likewise, the current algorithms are insufficient to overcome this knowledge barrier and enable advances in regenerative medicine, synthetic biology, and living machines/biobots. We propose a comprehensive conceptual framework for the engine of regeneration with hypotheses for the mechanisms and algorithms of stem cell-mediated regeneration that enables a system like the planarian flatworm to fully restore anatomical (form) and bioelectric (function) homeostasis from any small- or large-scale damage. The framework extends the available regeneration knowledge with novel hypotheses to propose collective intelligent self-repair machines with multi-level feedback neural control systems driven by somatic and stem cells. We computationally implemented the framework to demonstrate the robust recovery of both form and function (anatomical and bioelectric homeostasis) in an in silico worm that, in a simple way, resembles the planarian. In the absence of complete regeneration knowledge, the framework contributes to understanding and generating hypotheses for stem cell mediated form and function regeneration, which may help advance regenerative medicine and synthetic biology. Further, as our framework is a bio-inspired and bio-computing self-repair machine, it may be useful for building self-repair robots/biobots and artificial self-repair systems.

Significance statement Regeneration refers to the ability of an organism to maintain its form and function throughout its life. How regeneration happens is largely a mystery with many unanswered questions. This study presents a conceptual and computational framework for the machinery of self-repair in living systems that addresses some fundamental questions of regeneration to produce a system that accurately regenerates form and function after any damage or disturbance, much like the immortal planarian flatworm. The concepts and algorithms proposed in the framework have significant implications for the fundamental understanding of developmental biology and the maintenance of life, as well as for improving human health, longevity and biomedicine. Further, it can inspire new developments in biobots and robots not only in the fields of biology, health and medicine but also in other fields including engineering, computing, environment.

## Introduction

In biology, regeneration refers primarily to morphological processes that characterize the plasticity of the phenotype of traits that allow multicellular organisms to repair themselves (self-repair) and maintain the integrity of their morphology (form or anatomical pattern), and their physiological state (function). This is a process of collective action, through cellular computing in living systems in the form of soft robotics that displays a high level of complex adaptive decision-making. With body-wide immortality, Hydra (tiny aquatic animals) and planarians (free-living flatworms) are models of adaptive regeneration (1). After being injured, cells in any amputated fraction fully restore tissues and organs to their previous anatomical and physiological state. The regeneration of organs is widespread in animals such as snails, axolotls (an amphibian known as “walking fish”), and zebrafish (2). In a related context, some animals can reproduce asexually through fragmentation (e.g., starfish, some worms, fungi, plants, and lichens), budding (e.g,. yeast, Hydra), or fission (e.g., bacteria, protists, unicellular fungi) (3). For example, a planarian mother will narrow and split in the middle. Each half creates a new head or tail to form two copies of the original. Despite decades of research on regeneration, how animals reproduce and maintain their morphology and physiological state (e.g., bioelectric homeostasis) remains largely unknown. Living machines and soft robotics require testable models to explain the methods and mechanisms used to maintain the correct morphological and physiological state of the organism (4). This lack of knowledge has impeded the progress of regenerative medicine. This study proposes a conceptual framework for whole organism morphological and physiological regeneration of a simple synthetic worm modelled on planarian regeneration.

**Fig. 1.**
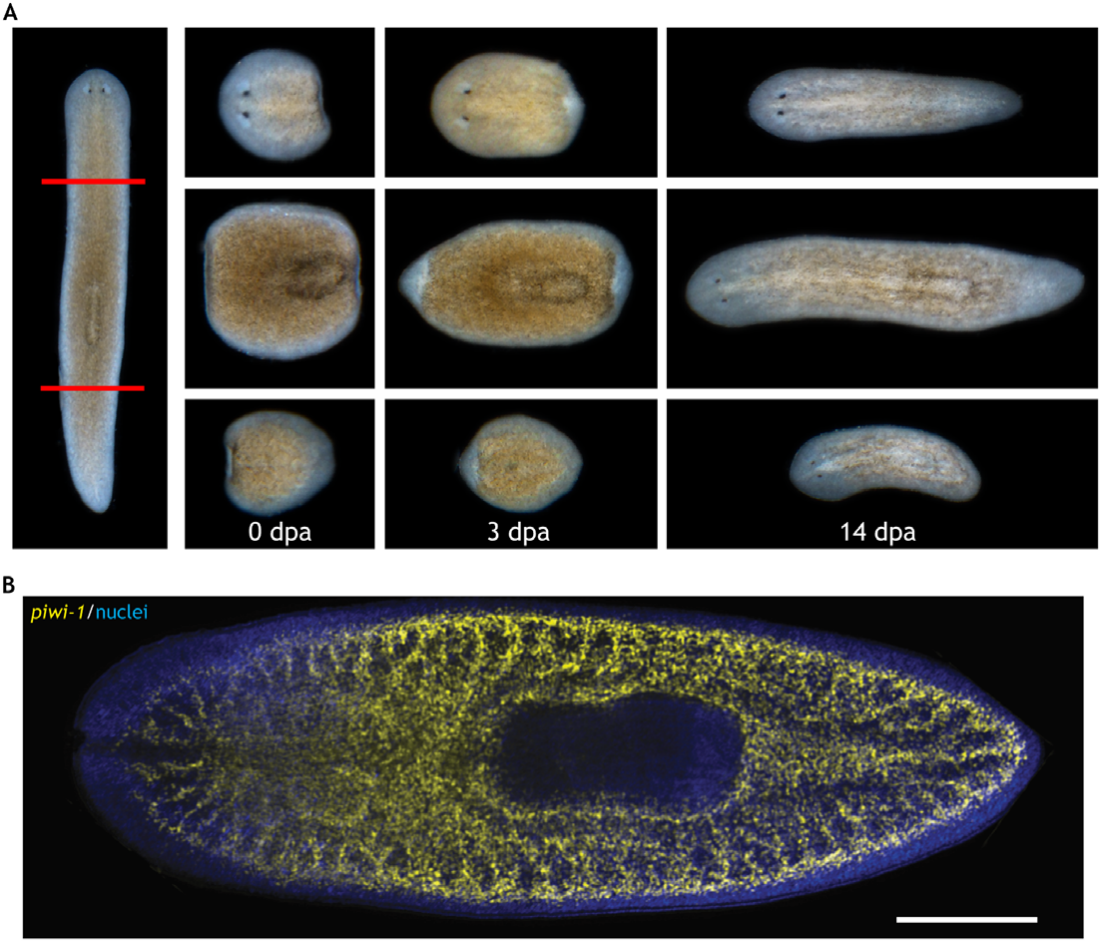
Planarian regeneration and the stem cell system. (A) Regeneration of the head (top), the body (middle), and the tail (bottom), showing the complete regeneration of individual parts into a full worm. Red lines indicate the cutting planes. Days postamputation (dpa) are observed time points. (B) The distribution of stem cells (yellow); blue indicates somatic (tissue) cells. Scale bar: 500 μm. (Source: (6), reproduced with permission. https://doi.org/10.1242/dev.167684).

A 6 mm long planarian (Fig. 1A) has approximately 0.6 million cells (Fig. 1B) (5), with adult stem cells (shown in yellow), estimated to account for as much as 20% to 30% of all cells, distributed across the planarian's body (6). Stem cells produce tissue (somatic) cells that, in the process of development of the organism, divide. After incurring damage, stem cells migrate to the location of damage and reproduce all necessary types of stem and somatic (tissue) cells to regenerate lost tissue and organs, completely restoring the organism. How stem cells (in collaboration with the remaining cells) achieve this remarkable recovery is unknown. In a planarian, a severed head, body, or tail takes just 2 weeks to grow into a fully formed planarian (Fig. 1A). Planaria are also capable of identifying anterior-posterior (A/P) (along the body) and Dorsal-Ventral (D/V) (across the body) polarity and patterning (form) information via bioelectric signaling (7, 8) meaning that a small piece of planaria can locate the new head and tail correctly as in a normal planarian (Fig. 1A) (9). How the remaining cells in a worm split into two segments re-establish polarity in the two segments during regeneration is still unclear. Scientists believe that stem and somatic/tissue cells communicate via bioelectricity, but exactly how this occurs is still unclear (10). A single stem cell, introduced after damage, can reproduce new stem cells that regenerate the whole pattern in an irradiated animal where no stem cells remain (11). While the body-wide immortality (ability to regenerate from any damage anywhere) of planaria and the process of regeneration in organisms has attracted much research interest, many questions about the mechanisms and algorithms of regeneration remain. Answering these questions may not only lead to new possibilities for human longevity, but also open avenues for designing advanced self-repairing robots and biobots that resemble living systems.

Understanding the “mechanisms” and the associated “algorithms” of regeneration is crucial for unravelling how organisms restore the correct anatomy and physiology of organs and the whole organism. In an organism, many physiological functions are sustained by bioelectricity (12). Upon successful regeneration, it is therefore crucial to maintain the membrane voltage of individual cells across an organism (i.e., body-wide bioelectric equilibrium or homeostasis). However, we still do not understand the mechanisms that coordinate cells into tissue nor how organs are arranged during regeneration. We also do not understand how bioelectricity modulates these responses nor how bioelectric homeostasis is restored by an organism after regeneration. This study provides a high-level conceptual soft robotic framework for the accurate regeneration of a simple organism. Using stem and somatic cells that communicate via bioelectric signals, the organism is able to restore both anatomical and bioelectric homeostasis. There are currently no computational models for the accurate maintenance and restoration of morphology (form) or physiology (e.g,. bioelectric homeostasis) or both.

Only a few researchers have attempted to create soft-robotics models of biological pattern regeneration using simple synthetic systems. Most have achieved limited success. Furthermore, no one has attempted the restoration of voltage homeostasis from perturbations under normal or damage conditions. Most of the past computational regeneration models have used simple (nonbioelectric) signal interactions: that is, between stem cells and somatic cells or only between stem cells in tissues (4, 13–15). These models range from simple ones that attempt to restore the total signal received by cells to agent-based (15) and neural network models (4, 16). Most of these models require too much information on pattern (form), interactions, computations, and rules for recovering the normal pattern for simple tissue structures. These systems do not fully recover and they do not know when to stop regenerating.

In recent decades, the number of studies on nature-inspired self-repair has increased significantly in robotics (17, 18), software (19–23), and electronics (24, 25). Most of these studies concentrate on function-only repair using information and communication within or without (i.e., internet). A few of these systems can restore form, but to a limited extent (they need new entities or spare parts from “outside”). This is a stark departure from biological systems that regenerate new cells from within (6). However, some of these self-repair models share similarities with the few existing biological regeneration models, the most prominent being short- and long-range communication between entities.

In our previous work, we addressed some of the unresolved issues in biological regeneration (26). We raised some fundamental questions about regeneration and provided hypothetical answers for regeneration in a simple tissue system. These questions included: how do stem cells sense damage and cooperate with somatic cells to repair local tissue damage? How do stem cells work together in large-scale repair? How do stem cells know what to recover and when to stop? Where could such morphological (pattern) information be stored? How could this information be retrieved during the process of regeneration? Importantly, how does a small body fragment regenerate into a fully formed organism (with correct polarity), to achieve body-wide immortality? In our previous article (26), we provided a simple soft-robotics computational framework to address these issues of regeneration as a collective intelligence problem. That framework, developed for a synthetic (in silico) worm consisting of three tissues (a head, body, and tail), was based on simple binary communication between cells, indicating their presence or absence, and a simple stem cell arrangement where there was only one stem cell per tissue with a large number of somatic cells. It was also not designed to restore the original function after regeneration.

Our new framework greatly extends the previous one's capacity by introducing new concepts to address deeper issues of regeneration in biological forms. This framework produces a soft robotic system that dynamically maintains both physiological and morphological homeostasis. It is more biologically realistic with respect to planarian anatomy; it has a greater number of stem cells. This framework also includes cellular communication via bioelectricity, which enables it to restore both form and function. This new framework raises further questions: how do the stem cells, dispersed throughout a tissue (Fig. 1B), accomplish form regeneration in collaboration with tissue cells? What communication structures are involved in this process? How does a cell collective hold a bioelectric pattern in the form of a body-wide voltage gradient? Finally, how does the organism restore bioelectric homeostasis under normal perturbations due to regular physiological functioning or in regeneration?

**Fig. 2.**
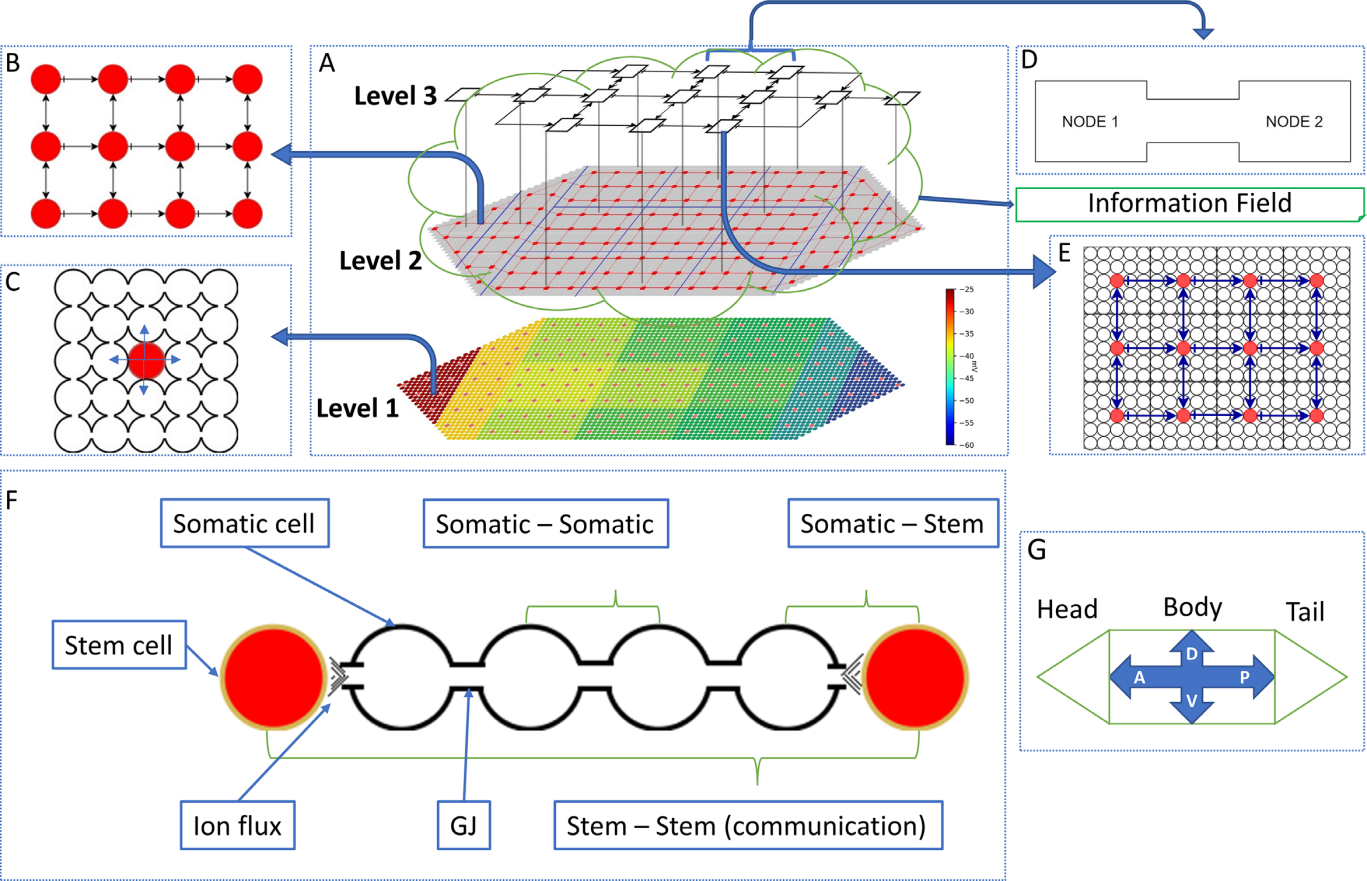
Detailed view of the framework for autonomous restoration of anatomical and bioelectric pattern homeostasis. (A) Three levels of the framework: level 1—the somatic/tissue cell network (the bottom layer). There are approximately 150 stem cells (red dots) distributed through the organism and 3750 somatic cells surrounding the stem cells (tiny dots filling the whole organism); colors indicate voltage, increasing from green to red as indicated in the scale bar to the right) (27). Level 2–the stem cell network (the middle), (the engine of regeneration) with an information field that contains minimum pattern and bioelectric information; and level 3—the global aggregate nodal network (the top). All three levels form computational networks: levels 1 and 2 are perceptron networks that collectively perform pattern regeneration; level 3 creates an associative memory network (AMN) (the top) that restores bioelectric homeostasis; all 3 levels cooperate in pattern and bioelectric homeostasis. (B) A detailed view of the stem cell network, showing connections between the stem cells. (C) The somatic cell network in a segment of tissue consisting of a stem cell (red) surrounded by somatic cells. These segments repeat to form the head, body, and tail tissues that constitute the whole pattern. (D) Two nodes of the AMN where the connection represents four Gap Junctions (GJ) connecting four somatic cells in the respective nodes. (E) A detailed view of an AMN node that comprises a segment of a tissue with approximately 300 cells: stem cells (red dots) and somatic cells (white dots). In all networks, arrows indicate the flow of bioelectric communication signals. (F) Three types of communication between cells (red and white cells are stem and somatic cells, respectively): communication between two somatic cells occurs through GJ; communication between a stem cell and a neighbor somatic cell occurs via ion fluxes; between two neighbor stem cells, communication occurs indirectly, through the GJs of intermediate somatic cells and ion fluxes. (G) The two main axes of the planarian structure: A/P (along the body) and D/V (across the body).

To better understand regeneration, our framework incorporates what is known and what is required for accurate regeneration and bioelectric homeostasis that resembles body-wide immortality in planaria. In this new framework, a change in the bioelectric state due to normal physiological functioning or damage, triggers both appropriate regeneration mechanisms and those that restore bioelectric homeostasis. Our conceptual framework integrates mechanisms and algorithms of pattern and bioelectric homeostasis within a multi-level organization of bioelectricity mediated computing cell networks (Fig. 2) that form a neural control system that autonomously maintains form and function under any small or large disturbances. We use a simple artificial (in silico) worm-like organism to demonstrate the accuracy and robustness of the regeneration framework.

The proposed soft robotic framework may enable the creation of self-repairing biobots, synthetic living machines, and artificial robots (28–33). It may also provide a realistic platform for advancing hypotheses to better understand the algorithmic mechanisms of regeneration and promote advancements in regenerative medicine for solving biomedical problems such as congenital disabilities, injury from trauma, cancer, and ageing (34).

Fig. 2 shows the framework, highlighting the concepts of regeneration—the mechanisms and algorithms—using a synthetic worm with three tissues, head, body, and tail (Fig. 2A). It contains novel aspects: first, we assume that the organism maintains a longitudinal bioelectric gradient, from the head to the tail, and a transverse bioelectric gradient, across the body (Fig. 2A level-1) (7, 8). Second, the organism has a larger number of stem cells (4% of the total) distributed throughout the body (Fig. 2A, level-2) (15). (We use 4% to test the regeneration concepts but a larger proportion of stem cells can be added to the framework in future). Third, all communication follows the body-wide bioelectric gradient. Fig. 2F provides an example of a local neighborhood of stem cells consisting of two stem cells (red color) and four intermediate somatic tissue cells. Tissue cells communicate with each other locally and directly through physical connections called GJ. Stem cells communicate with (neighboring) somatic cells via ion fluxes released to the environment. Accordingly, stem cells communicate with each other indirectly through the GJs of intermediate somatic cells and ion fluxes (Fig. 2F).

Our conceptual framework integrates three structural levels of the organism—the somatic cell structure, the stem cell structure, and a global (aggregate) cell structure (Fig. 2A)—and their mechanisms of interaction needed to restore anatomical and bioelectric homeostasis. The stem cell network is the engine of regeneration (Fig. 2A level 2 and Fig. 2B). Somatic cells form networks with local neighborhood communications (Fig. 2C), represented by perceptron neural networks with local interactions, in each of the three tissues (head, body, and tail) of the organism. Stem cells form a similar, but one body-wide, perceptron network. In both networks, perceptrons communicate their bioelectric state with their neighbors using three simple generic communication motifs. These motifs were trained to represent the neighborhood rules applicable to the three possible locations of a cell (interior, corner, or border) in the respective networks. They identify the presence or absence of neighbors and thus simplify body computing. The two perceptron networks identify missing neighbors based on changes in the neighbor's bioelectric state. Global nodes form a 13-node network represented by an AMN (a modified form of a Hopfield network) (Fig. 2A—level-3 and D) that recognizes and restores the body-wide homeostasis bioelectric pattern of the nodes after any perturbation or damage. These nodes consist of a cluster of stem cells surrounded by somatic cells (Fig. 2E) that together constitute the organism. The AMN is trained to store the homeostasis bioelectric pattern in its attractor. Further, an information field containing a minimal body plan for the tissues [tissue length (d), the aspect ratio (AR) (length/width), the number of corners (n)] and the homeostasis bioelectric state surrounds the organism and is accessed by stem cells and the AMN. Fig. 2G shows the two main axes of the organism, A/P and D/V, respectively.

The "Methods" Section provides the organizational and functional description of the framework that features five stages (Fig. 7A and B) of regeneration. These stages cover how somatic and stem cell networks detect a bioelectric state change anywhere in the system through the changes in their neighbor's bioelectric state; how perceptron communication then identifies whether the change is due to normal function or damage; and, in the case of no damage, how the AMN restores bioelectric homeostasis; and, in the case of damage, how perceptron communication identifies the missing neighbors in the somatic and stem cell networks depending on the damage; how neighbor stem cells of damaged stem cells regenerate new stem cells that migrate to the damage site in the somatic cell network and completely repair the damage with the help of the somatic cell network; and how the stem cell network subsequently informs the AMN that incrementally restores bioelectric homeostasis by updating the nodal voltage until it matches the voltage pattern stored in its attractor. The Methods section also describes how the AMN, the stem cell, and the somatic cell networks are trained to accomplish these tasks.

## Results

### Implementation of the framework for automated pattern regeneration and bioelectric restoration: organism maintains body-wide immortality and bioelectric homeostasis

In this section, we demonstrate the implementation of the framework on a simple synthetic (in silico) worm to show that it efficiently and robustly recovers from both very simple to very complex damage. We consider two main types of damage: partial tissue damage with intact stem cells; and severe damage incurring the loss of stem cells and tissue segments or whole tissue (head or tail). We illustrate how the framework efficiently restores the bioelectric pattern due to normal perturbation or damage.

### The model worm and its original geometric pattern and bioelectric state

The artificial model organism is a simple worm with head, body, and tail tissues consisting of 150 stem cells (red dots) distributed throughout the organism. There are 3750 somatic cells surrounding the stem cells (tiny dots that fill the whole organism) (Fig. 2A). The organism consists of repeated patterns (square blocks) of stem cell-centred somatic cell neighborhoods, sized 5 × 5 (Fig. 2C) (24 somatic cells per stem cell). The arrows in Fig. 2B and E and Fig. 2C respectively, show the closest neighbors of the stem cells and the somatic cells. The colors in Fig. 2A show the body-wide bioelectric gradient, indicating decreasing cell membrane voltage, from the head to the tail, and from the middle of the body to the border regions (7, 8). The homeostasis bioelectric pattern is negative throughout the planarian indicating that the membrane voltage is hyperpolarized.

### Organism restores body-wide bioelectric pattern due to normal perturbation (without damage)

The AMN refers to body-wide associative memory. It is a mechanism that helps its nodes communicate, through links with trained connection weights, to collectively maintain the voltage in their constituent cells following any perturbation. The AMN is trained with Hebbian learning using a large number of perturbed voltage patterns of nodes, representing changes due to normal physiological function and damage. The trained network remembers the original bioelectric pattern in its attractor and stores it in the information field (see the "Methods" section). Changes to nodal voltage triggers the AMN to restore the original bioelectric pattern.

**Fig. 3.**
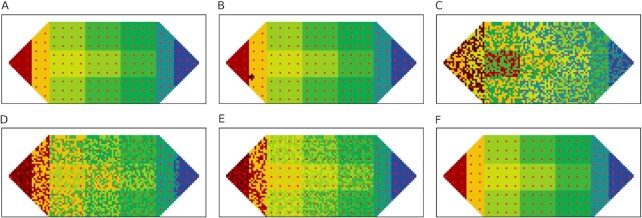
Perturbation and restoration of body-wide bioelectric voltage pattern under normal physiological function. (A) Normal equilibrium voltage pattern (homeostasis); (B) One or a few cells in a global node experience a voltage change—up to ± 10%; (C) All or most of the cells in all nodes experience voltage change—up to ± 10%; (D&E) Stages of recovery of the bioelectric state via activation of the AMN; (F) Recovered original voltage pattern.

In the resting condition, the original body-wide bioelectric pattern takes the form of Fig. 3A. When a cell or a few cells experience a change in voltage, the corresponding AMN node *k* that the cells reside in also undergoes a change in its voltage ***V_k_*** (Fig. 3B). This change triggers the AMN network to restore the original bioelectric pattern of the affected nodes and cells (Fig. 3F). This case follows Stages 2→5→1 of the regeneration framework (see Fig. 7B). These stages are: detection of bioelectric change and confirmation of no damage (stage 2), restoration of the bioelectric state (stage 5) and a return to monitoring (stage 1). When somatic cell(s) experience a voltage perturbation, stage 2 (Fig. 7B) is activated to determine if this change is due to normal functions or damage. Voltage changes due to normal physiological functions are typically below 10% (35). The affected cells in the somatic cell network send bioelectric signals through the GJs and ion fluxes to the stem cells. Both the stem and somatic cell networks detect this change. The affected somatic and stem cells check for missing neighbors by applying the three perceptron communication motifs for the corner, border, and interior cells (see the "Methods" Section and Fig. S1). These perceptron motifs are trained to take the neighbors’ voltage as input and predict their presence or absence. In this case, the affected cells find that there are no missing somatic or stem cells, and the stem cell network instructs the AMN to restore the bioelectric state (stage 5). Beginning with the altered voltage pattern as input, the AMN restores the original voltage pattern stored in the attractor in a series of steps. This process of gradual restoration allows cells to incrementally produce the required changes in voltage. The AMN remains active until the system has returned to the original bioelectric state (Fig. 3F) (stage 1). Even if a greater number of (or all) cells experience deviations from the equilibrium voltage state (see Fig. 3C), the AMN similarly restores the original bioelectric pattern in a series of steps (Fig. 3D to F). The framework thus restores bioelectric homeostasis due to any physiological disturbance.

### Organism recovers from small or large scale damage

#### Case 1: (a) framework recovers from simple damage with one somatic cell missing

**Fig. 4.**
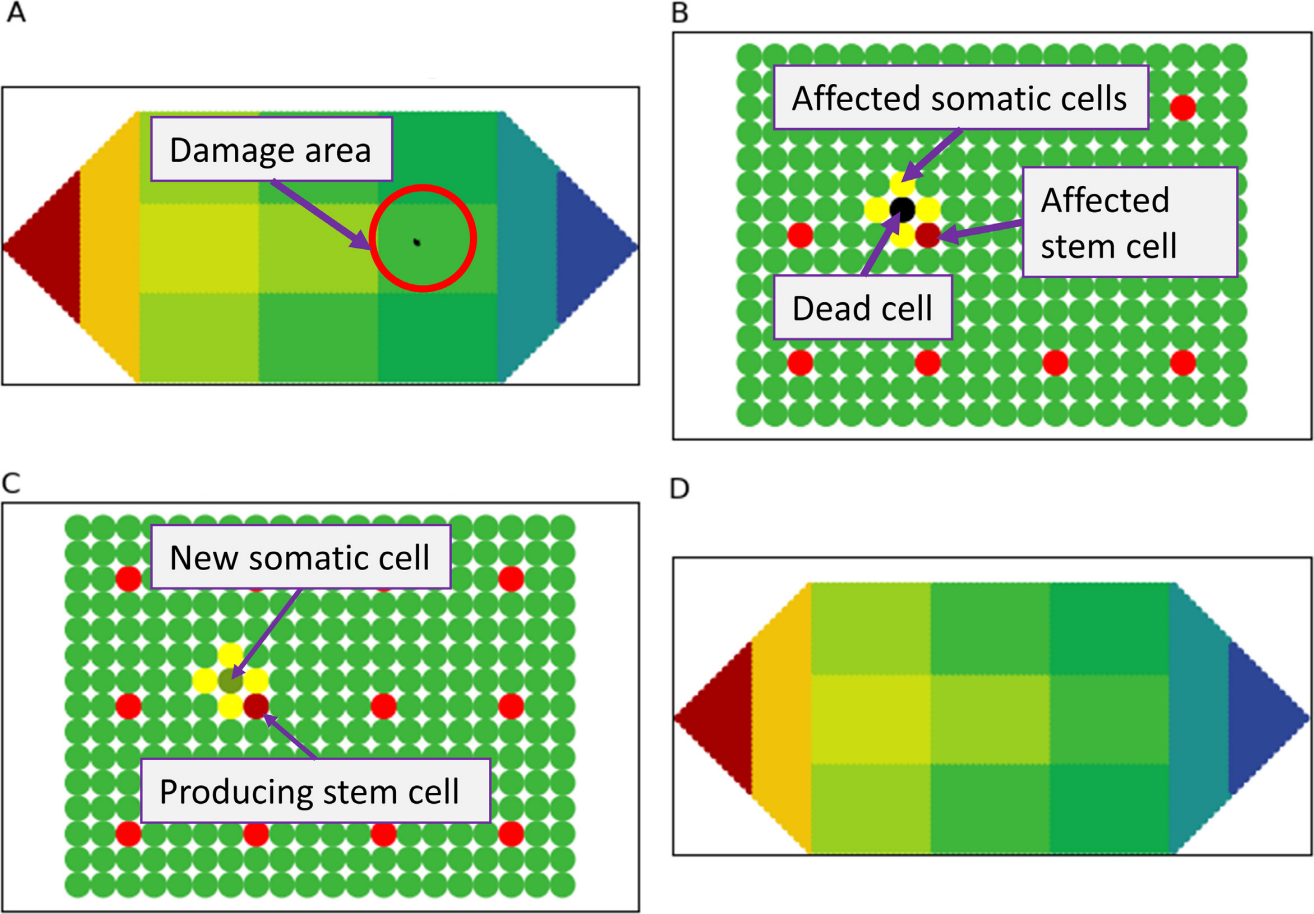
An example of a somatic cell damage and its regeneration and bioelectric state restoration. (A) One damaged somatic cell (black cell); solid circle highlights the affected cell area; (B) Damaged region showing neighbors (yellow cells) of the damaged cell. The red cells are stem cells in this tissue; the dark red cell is the nearest stem cell affected by the damage; (C) regeneration of the missing cell by the affected stem cell (the producing stem cell); the regenerated cell has the voltage of the producing stem cell; (D) AMN recovers the equilibrium bioelectric pattern.

Here cell death occurs due to ageing or harmful factors such as trauma or toxic chemicals (Fig. 4A and enlarged in Fig. 4B). This case involves simple damage where only one somatic cell is missing anywhere in the organism. This damage case helps illustrate some fundamentals of the framework simply. When a somatic cell dies, connections between the damaged cell and its neighbor somatic cells through the GJs are lost. The neighbor cells’ voltage increases by > 10% (35) due to the electrolytes released from the dead cell. This process activates Stages 2→3→4→5→1 of the framework (Fig. 7B). These stages are: detecting bioelectric change and confirming damage (stage 2), identifying damage (stage 3), restoring damage (stage 4), restoring the bioelectric state (stage 5) and returning to monitoring (stage 1). The somatic cell network experiences the voltage change and sends bioelectric signals through the GJs and ion fluxes to the stem cells (stage 2). After sensing the increased voltage, indicative of damage, the affected somatic cells apply the three perceptron communication motifs to confirm that damage has actually occurred (stage 3). The cells with missing neighbors change their status to “damaged.” As illustrated in Fig. 4B, the “damaged” somatic cells (yellow cells) are neighbors of the damaged cell (the black cell) and become the damage border. The affected stem cell assesses the neighboring stem cells using the relevant perceptron motifs and determines that all of its neighbor stem cells are intact. Here, only one somatic cell is lost.

In regeneration (stage 4), the affected stem cell (the dark red cell in Fig. 4B) moves to the damage border and divides to produce a new somatic cell. The new cell's voltage differs from that of the original cell and, in the absence of clear evidence, is assumed to be similar to the voltage of the stem cell that produced it (Fig. 4C); in reality, it can either be this or between the original voltage and that of the somatic cells in the damage border. After regeneration, the new cell establishes connections with its neighbors and the relevant neighborhood rules and corresponding communication motifs. All cells in the damaged border recheck their neighbors and update their status. As all these cells now have the correct number of neighbors as in the initial state, their status returns to “normal.” The stem cell returns to its original place in the tissue and activates the AMN to restore the bioelectric state (stage 5), similar to the case without damage. The increased voltage in the cells in the damaged area (yellow and dark red cells in Fig. 4C) results in a slight increase in the corresponding AMN node's voltage. Upon activation, the AMN uses the bioelectric state after regeneration as input and, in a series of steps, returns the altered nodes’ voltage to the normal state (Fig. 4D).

#### Case 1: (b) framework recovers from damage, with multiple missing somatic cells

Successful regeneration and restoration of bioelectric homeostasis after damage to multiple somatic cells is presented in section S3, case 1 in Supplementary Materials and Fig.S4.

#### Case 2: (a) organism recovers from damage involving a stem cell and its surrounding tissue

**Fig. 5.**
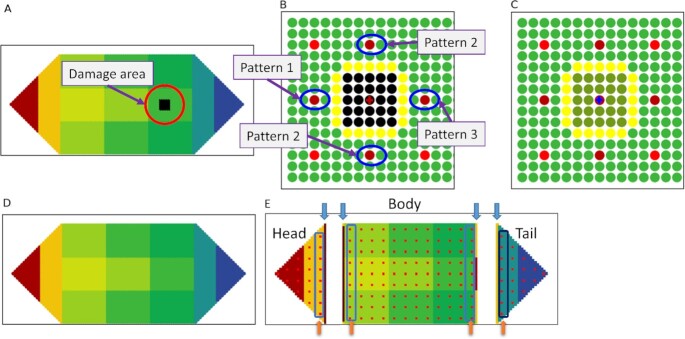
Damage involving a stem cell and surrounding tissue and its regeneration and bioelectric state restoration. (A) A stem cell and surrounding tissue damage (black cells); (B) identification of damage—yellow cells show the damage border of the somatic cell network. The dark red dots indicate the damage border of the stem cell network. The middle black cell with a red cross in the damage region indicates the missing stem cell; (C) regenerated missing stem cell (red cell with a blue cross) and somatic cells and the bioelectric state after regeneration; (D) The AMN restores the body-wide bioelectric pattern; (E) damage to whole body parts—the worm is completely cut into three separate tissues: head, body, and tail. Blue arrows indicate the somatic cell damage borders identified by the respective tissue somatic cell networks in the three tissues. Orange arrows indicate the stem cell damage borders identified by the stem cell network.

This example is a more complex damage case where a stem cell and a considerable number of somatic cells (about 24 cells) are gone (see Fig. 5A). The damaged region is highlighted in Fig. 5B (black cells) to show the missing stem cell and its surrounding tissue. As in case 1, somatic cells neighboring the damaged cells first detect the bioelectric change and alert the stem cell network (stage 2). Since the voltage change is greater than 10%, they attempt to identify the damage (stage 3). Neighbors of the damaged cells establish the somatic cell damage border (the yellow border in Fig. 5B) using the relevant perceptron motif (in this case, for an interior cell). Fig. 5B shows that the four nearest stem cells (the dark red cells) experience an altered bioelectric state and they check their stem cell neighbors using the relevant perceptron motif (for interior cells). These four stem cells establish the stem cell damage border and identify that one neighbor stem cell is missing. To identify the nature and size of damage, three pattern primitives (see the "Methods" section) are applied to the stem cell damage border to determine if the damage is enclosed, has open borders, or whole tissues are missing.

The three primitives are basic patterns that help identify the nature of the stem cell damage by assessing if there are border stem cells (i) to the left, (ii) above or below, or (iii) to the right of the damage, representing pattern primitives 1, 2, and 3, respectively (see Fig. 5B). For example, if all patterns apply, the damage is local (encased or with an open border). If only some of the patterns apply, there is large-scale damage; for example, if only one pattern applies, this indicates complete tissue damage. In Fig. 5B, all three patterns apply, as there are border stem cells to the left, right, and above and below the damage, indicating local encased damage. To regenerate (stage 4), any stem cell in the border can migrate to the (yellow) somatic cell damage border and begin the regeneration process. The stem cell first produces a new stem cell with the same voltage as itself. The new stem cell then produces new tissue with 24 somatic cells, guided by the somatic cell damage border, to achieve accurate regeneration. In this case of interior damage, new somatic cells fill the (black) damage region, at which point regeneration stops (Fig. 5C). To restore voltage (stage 5), the stem cell network activates the AMN to revert to the original pattern (Fig.5D).

#### Case 2: (b) organism recovers from damage involving multiple stem cells and surrounding tissue—one whole AMN node missing

Successful regeneration and restoration of bioelectric homeostasis after damage, involving multiple stem cells and surrounding tissue is presented in section 3 case 2 in Supplementary Materials and Fig.S5.

#### Case 3: (a) organism recovers from damage to whole body parts—completely missing head, body, or tail

##### Sensing damage and identification of lost tissue/s

In this last example, the worm is cut into three parts: the head, body, and tail (Fig.5E). As described in the previous examples, stem cells and somatic cells in the severed parts sense the voltage change (stage 2). First, somatic cells identify the border of the tissue damage (blue arrows in Fig. 5E) using perceptron motifs. The affected stem cells check their neighbor stem cells using the perceptron motifs. This process establishes the border of stem cells in each tissue; specifically, four stem cell borders (one each for the head and tail and two for the body) (orange arrows in Fig. 5E). The stem cells identify the severity of damage (Stage 3). Applying the three pattern primitives to the actual stem cell borders reveals that these border stem cells encompass only one generic pattern primitive (i.e., border stem cells found either to the left or right of damage), which means whole tissue damage. Border stem cells then identify the type of missing tissue (head, body, or tail) by applying two specific rules (see the "Methods" and Section S1.3 in Supplementary Materials) that recognize the direction of disrupted bioelectric communication in the worm:

Severed head tissue: the border stem cells in the head tissue do not receive any signals along the AP direction (from the body side). They still receive normal signals (from the head side), so they recognize that the body is lost.

Severed body tissue: for the damaged side on the left: stem cells do not receive signals along the AP direction (from the head side). They receive signals from the tail side; therefore, they recognize that the head is lost. Similarly, for the damaged side on the right: stem cells do not receive signals along the AP direction (from the tail side). They still receive normal signals (from the head side), so they recognize that the tail is lost. This indicates that the body tissue is separated.

Severed tail tissue: the stem cells nearest to the damage border identify the type of damage as the head and body missing: these stem cells do not receive signals along the AP direction (from the head side). They still receive normal signals (from the tail side).

All three separated tissues regenerate (stage 4) into three separate worms. The case of the head regenerating the whole worm is described below.

##### Regeneration: head regenerates body and tail

**Fig. 6.**
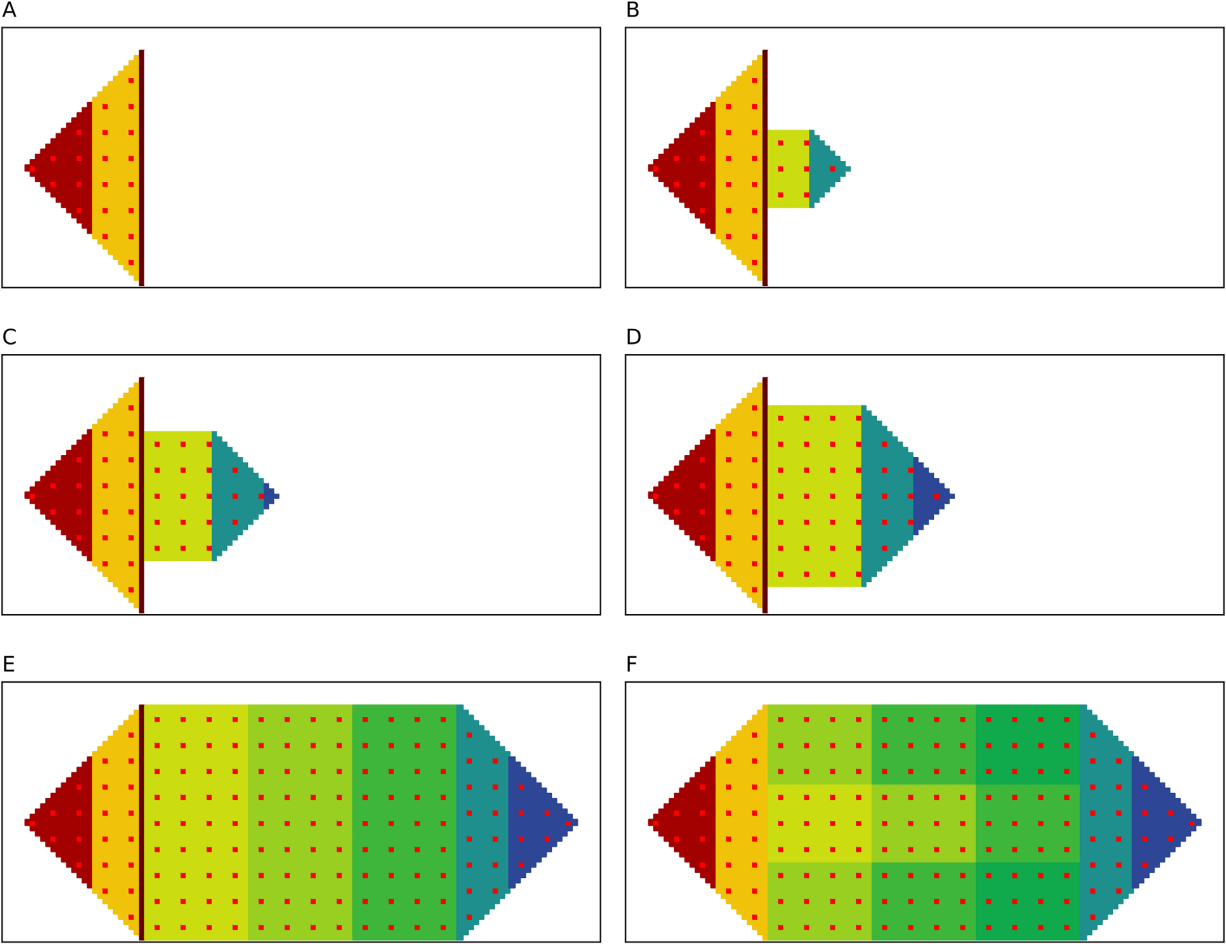
The head regenerates the body and tail to restore the whole form and bioelectric pattern. (A) Severed head tissue showing the stem cell border close to the damage site; (B to E) regeneration of the body and tail stem cells by the stem cells in the stem cell border and concurrent regeneration of head and tail tissues until full recovery of anatomy; and (F) AMN restores the body-wide bioelectric pattern.

Stem cells in the damaged border (Fig. 6A) produce new stem cells for the body and the tail and transfer respective minimal tissue shape information from the information field (AR, d, n) to them. They then produce their respective tissues (body and tail) concurrently (Fig. 6B). The new cell's voltage is lower than the producing stem cells due to the bioelectric gradient from head to tail. The small body and tail grow incrementally into the exact original form following the shape information obtained from the information field (Fig. 6B to E). The bioelectric pattern (soon after regeneration) is shown in Fig. 6E. Finally, the AMN restores its network configuration and retrains the weights using the original bioelectric pattern stored in the information field. This way, it restores the repaired nodes’ voltage (stage 5) and brings the whole worm back to the equilibrium voltage pattern (Fig. 6F) (stage 1). The severed body and tail also regenerate the whole worm using the same process.

#### Case 3: (b) organism recovers from damage to whole tissue along with interior damage

Successful regeneration and the restoration of bioelectric homeostasis after this type of complex damage is presented in Section S3 case 3 in Supplementary Materials (Figs.S6 to S8).

#### Case 4: organism recovers from more complex damage where worm is split into two with vertical and horizontal cuts

Successful regeneration and the restoration of bioelectric homeostasis after this type of complex damage is presented in Section S3 case 4 in Supplementary Materials (Figs. S9 to S11).

## Discussion

In this study, we asked whether it is possible to conceptualize the mechanisms and algorithms of regeneration of an amazing model organism like the planarian. These organisms achieve body-wide immortality and maintain bioelectric homeostasis, which enable them to continue functioning perpetually. To answer this question, we proposed an autonomous computational soft robotics self-repair framework that displays body-wide immortality and restores bioelectric homeostasis, much like the planarian, in a simple in silico worm. We demonstrated the application of the framework to successfully detect and completely and accurately repair diverse forms of damage and restore bioelectric homeostasis. Our framework is an intelligent control system within a neural computing paradigm for collective cellular decision-making for the dynamic restoration of morphology and physiology. The model combines three levels: somatic cell networks and a stem cell network, both represented as locally interacting perceptron networks for damage identification and full and accurate recovery from diverse forms of injuries, and a high-level AMN, for the restoration of body-wide bioelectric homeostasis. We demonstrated the efficacy in recovery using several very small to very complex damage cases through the implementation of the framework. The three networks function collectively to maintain the anatomical pattern and bioelectric homeostasis. We believe that this is the first work that provides a conceptual basis and algorithms explaining how an organism like the planarian can maintain and restore form and function.

In this framework, bioelectricity is a fundamental property of the cells. It helps cells communicate with each other to maintain the original form and body-wide bioelectric gradient. The somatic cell networks transfer bioelectric signals to the stem cells and identify the border of tissue damage that guides the stem cells to injury location. As a body-wide network, stem cells are the engine of regeneration. As a result of bioelectric signaling between somatic and stem cells, stem cells can detect small local damage or complex large-scale damage involving lost stem cells or whole tissues and repair the damage completely and accurately with the help of the somatic cell networks. The AMN mimics an unknown mechanism in planaria that maintains the body-wide bioelectric gradient along and across the worm.

Our study contributes a comprehensive soft robotics framework to generate hypotheses about the mechanisms of regeneration to advance the knowledge in this field. It also contributes several concepts and algorithms to significantly advance computational models of regeneration involving minimal computation. We compare our model to the state-of-the-art regeneration models to demonstrate its novelty and efficacy.

Our previous regeneration framework (26) is the closest to the current work. The previous framework regenerated an organism of three tissues using binary communication between cells without using bioelectricity. We have greatly improved our previous study. First, we have proposed a greater number of stem cells, about 4% of the total, distributed within tissues. In the previous framework, only one stem cell represented each of the three tissues. Second, we introduced two types of bioelectric communications—through GJ and ionic fluxes. Somatic cells use physical GJs to connect to and communicate with adjacent cells. Stem cells maintain long-range bioelectric communications propagated through the intermediate somatic cells (ion fluxes moving through GJs and then released into the surrounding environment). While stem cell communications are contained within a local region, minimizing computation, the stem cell network is distributed throughout the organism. The current model is more biologically realistic compared to our previous one (26) where we used direct, long-range interactions by which the stem cell in the head can communicate with the stem cell in the tail. Third, we used an AMN as a mechanism to help the worm maintain the bioelectric gradient in an equilibrium state. We found an intriguing minimal network configuration for the AMN (with tail-to-head inhibition and head-to-tail activation) that efficiently stores and retrieves the equilibrium bioelectric pattern of 13 macro (global) nodes, each consisting of approximately 300 somatic cells and 12 stem cells (Section S1.4, Supplementary Materials). This discovered network configuration points to the possible existence of simplified and directional networks as opposed to fully connected networks within cellular structures that perform complex tasks such as the restoration of body-wide homeostasis. This structure helps the worm maintain the bioelectric gradient in the A/P and D/V directions. We established similar directional activation and inhibition in our configuration of the stem cell network to conform to the AMN (Fig. 2B).

Finally, we used three perceptron motifs, which are trained for the stem and somatic cell networks so that they can determine the missing neighbor stem and somatic cells. For the stem cell network, which is an organism-wide perceptron network, the weights of these motifs represent the effective conductance between two stem cells, and correspond to the conductance of the GJ of intermediate somatic cells and ion fluxes in living multicellular structures. For the tissue-specific somatic cell networks, the weights of these three motifs represent the bioelectrical conductance of the GJs between somatic cells. In the previous framework, we represented stem cells using a linear neuron with fixed weights and a somatic cell using a perceptron with fixed weights (equal to 1). In the new framework, the three motifs use cell voltage as inputs to communicate output for a somatic or stem cell located anywhere in the organism through trained network weights. The two cell networks collaborate to repair damage. The AMN then restores the bioelectric gradient. To identify various types of stem cell damage, ranging from one or a few stem cells to completely missing whole tissues, we proposed three simple pattern primitives applied to the stem cell damage border. In the case of missing whole tissues, we additionally proposed two simple rules involving the direction of the disrupted communication flow, from head to tail or vice versa, to identify the exact missing tissue.

Other prior models have attempted single or multiple tissue regeneration and have achieved varying degrees of success via a large amount of computation. None has achieved complete and accurate regeneration or presented a comprehensive framework. None has attempted bioelectric restoration. Bessonov et al.’s (13) tissue regeneration model assumes that an individual cell communicates with all other cells and remembers the total signal intensity value it receives and some regeneration rules. Regeneration is based on restoring the total signal intensity of each cell. However, there is a high computational burden due to the extensive communication between cells. The model also has limited success in accurate recovery. Due to the simple local communications, the computational burden in our model has been drastically reduced, while achieving complete and accurate recovery. In Tosenberger et al.’s (14) circular tissue model, regeneration is accomplished via stem cells that are not damaged. Our framework applies to multiple tissue shapes and allows stem cell damage. The stem cell network replaces missing stem cells. In their model, tissue regeneration requires all cells to compute the received signal intensity and compare it to a threshold value, which is computationally burdensome. Further, it assumes that the tissue has a survival region and that cells produced outside of this region are killed to achieve the required shape, which is biologically unrealistic. In our framework, local communication in somatic perceptron networks in tissues identifies the damage boundary, which guides complete and accurate recovery. In extreme cases involving tissue loss, minimum body plan information is accessed from the information field.

De et al.’s (4) model is one of the more biorealistic models. It involves interactions between a neural network (representing the organism's nervous system), and nonneural cells (representing tissue) to recover from damage using physiological variables. It has achieved reasonable regeneration success (55% to 80%), depending on damage severity. However, it does not recognize damage and generates and kills many cells before achieving a partially recovered form. Our framework detects damage and accurately recovers the complete form using simpler and more efficient computations in stem and somatic cell networks. As long as a single stem cell exists, our model can regenerate the whole organism correctly. Ferreira et al.’s (17) agent-based models require much communication between cells to discover cell structure and store much data. Further, these models cannot regenerate larger sized tissues as information can only go a limited distance or signals decay with distance. By contrast, the information/data our framework carries in regeneration is high-level and generic, with three simple perceptron communication motifs. It contains few rules relating to the stem cell damage border pattern and a minimum amount of information on shape to achieve efficient regeneration in any sized tissue. Importantly, it involves minimum computation.

### Future directions for the regeneration framework

Although our autonomous framework has emulated important aspects of planarian regeneration, other aspects can be incorporated into it to realize its ultimate goal of representing complete regeneration involving collaboration among all levels—molecular, cellular, tissue/organ, and whole system. From a conceptual perspective, there are two main challenges to understanding regeneration. One concerns the high-level view of how the whole process of regeneration takes place and the other concerns the detail view of how cellular and molecular processes support the whole regeneration process. Since our understanding of all these levels are incomplete, our study looked at it from the high-level functional view. However, we formulated the framework in such a way that it can not only be expanded in function but also information at all levels can be seamlessly integrated into the framework. This could make the framework a multi-scale representation of regeneration. This section provides some specific potential directions for its future.

From a functional perspective, the form of the chosen organism is simple and needs to be extended to more realistic shapes with external and internal features like the real planarian or other organisms. Further, our 2D model can be easily extended to 3D. For example, Fig. 1B, which presents the cross section of the planarian showing the distribution of stem cells across the organism gives a glimpse of its 3D stem cell configuration. We developed the 2D model to test the concepts. But our framework can integrate 3D configuration of stem cells with the same processes as in the original framework for regenerating 3D tissues. Also, cut pieces in our model recover a fixed geometry after any damage. Formation of altered forms as in Fig. 1A can be explored in the future. From a cellular perspective, the chosen voltage pattern broadly represents that of planaria, but it is coarse as the voltage of somatic cells in each AMN node is assumed constant. These cellular voltage values could be made to vary within a node to achieve a more refined and realistic bioelectric gradient. To make it even more intrinsic, the cellular voltage can be simulated by partial differential equation models of voltage patterns produced by bioelectric communications between adjacent cells through GJs (as in (27)). Our framework can integrate these cell-level bioelectric models in the future.

Further, our framework can incorporate models of molecular signaling pathways to activate biological processes to represent both cellular and molecular levels of regeneration. In this regard, the whole genome of the planarian has been recently assembled (36). Although many genes that are likely to play crucial roles in planarian regeneration still await functional characterization, some genes and molecular pathways involved in regeneration have been elucidated. A 2022 review (37) reports on some genes and molecular mechanisms involved in different cell types (eye spot, epidermal, muscle etc.) during planarian regeneration. For example, the extracellular signal-regulated kinase (ERK) pathway regulates stem cell differentiation and the Hippo pathway has been shown to play an important role in cell cycle regulation, tissue development, and organ size in planarian regeneration (37). An answered question is how the Hippo pathway controls organ size, which is a patterning issue. As cell division is a key aspect of regeneration, cell cycle molecular pathways can be incorporated into our framework in places where cell proliferation takes place including neoblast proliferation into tissue progenitors and their proliferation in turn into different somatic cell types. Davenport et al. (38) state that neoblasts from different anatomical positions display unique patterns of gene activity. With the representation in our framework of tissue-specific stem cells, molecular mechanisms of tissue specific neoblasts can also be incorporated into it.

Additionally, once we know more about the molecular processes involved in the regeneration of specific tissues in planaria, these can be incorporated into regeneration of specific tissues in our regeneration framework. Grohme et al. (36) state that some of the genes required for regenerating a head are already known and, with the assembly of the whole planarian genome, it is now possible to search for the mechanisms that control head genes only at the front end of a regenerating piece. However, they caution that a function-centric approach, rather than gene-centric approach, will ultimately facilitate reverse engineering in biology—this was also the view in developing a conceptual basis for the functional representation of regeneration in our framework.

In the absence of required knowledge, it is also possible to cross-fertilize our framework with the understanding of regeneration in other model systems. For example, Nowoshilow et al. (39) identified a number of transcripts that are upregulated in the limb blastema (the mass of proliferating cells involved in regenerating the limb) of axolotl. Some of these observations could correspond to planarian regeneration. Mulder et al. (40) say that the fate or role of the blastema cells seems to be intrinsically determined in that when transplanted to another site it regenerates according to its level of origin. This is also an interesting aspect that can be incorporated into our framework computationally by artificially transplanting stem cells corresponding to one specific tissue pattern into another tissue. Davenport et al. (38) interestingly note that regenerating cells need instructions (tutoring) and that regeneration studies will likely provide insights into the tutoring that cells need toward reconstructing entire organs.

Another promising aspect that could be incorporated into our framework is electrophysiological stimulation in repair and regeneration. Amazing results have been reported on the activation of regeneration in immobilized and irradiated planaria through direct current stimulation (DCS), which restored stem cell features that had been eliminated by radiation (41). This was accompanied by reactivation of cell cycle gene expression and DNA-damage repair response in lethally irradiated tissues. These findings show that electric stimulation can invoke molecular responses involved in repair and regeneration. As our framework is already based on bioelectricity, it is highly amenable to the integration of bioelectric stimulation in tissue regeneration. This could be an exciting addition to our framework.

A related interesting prospect for the framework will be the emerging roles of sulphated glycosaminoglycans (sGAG) as electro-regulatory mediators of intrinsic repair processes. GAG are biomolecules found in the glycocalyx and extracellular matrix (ECM) of all cells and involved in a wide variety of cellular processes during tissue development and remodeling (42). Charge transfer properties of sGAGs make them able to act as electrical conduits from the ECM to cells during electro-stimulatory applications and thus offer a potential mechanism for promoting cellular tissue repair processes. According to Hayes and Melrose (42), GAGs have been subjected to heightened evolutionary pressure to develop recognition and effector roles. They state that the inherent charge transfer and storage properties of GAGs make them in reality a bioinformatics “glyco-code” network with a language that cells can interpret, thus making it a comprehensive bio-IT database, which nature has developed over a very long evolutionary period. Hayes (43) has found an abundance of sGAG in two types of flatworms and according to Yamada et al. (44), synthesis and deposition of GAGs in the basal lamina of the regenerating planarian have been observed in 1980s. It is possible that GAGs in Planaria also have some signaling role relaying charge around the worm that directs cellular activity and stimulates regeneration. Therefore, cracking the glyco-code and embedding its language in our framework could provide a pathway for the flow of bioelectric and other regeneration related information between cells during regeneration. All these additions will enhance the framework's collective intelligence and decision-making capacity by capturing the fundamental processes involved in regeneration so that the system more accurately resembles living planaria and living systems. It could potentially help in enhancing and even activating healing and regeneration in humans.

## Methods

**Fig. 7.**
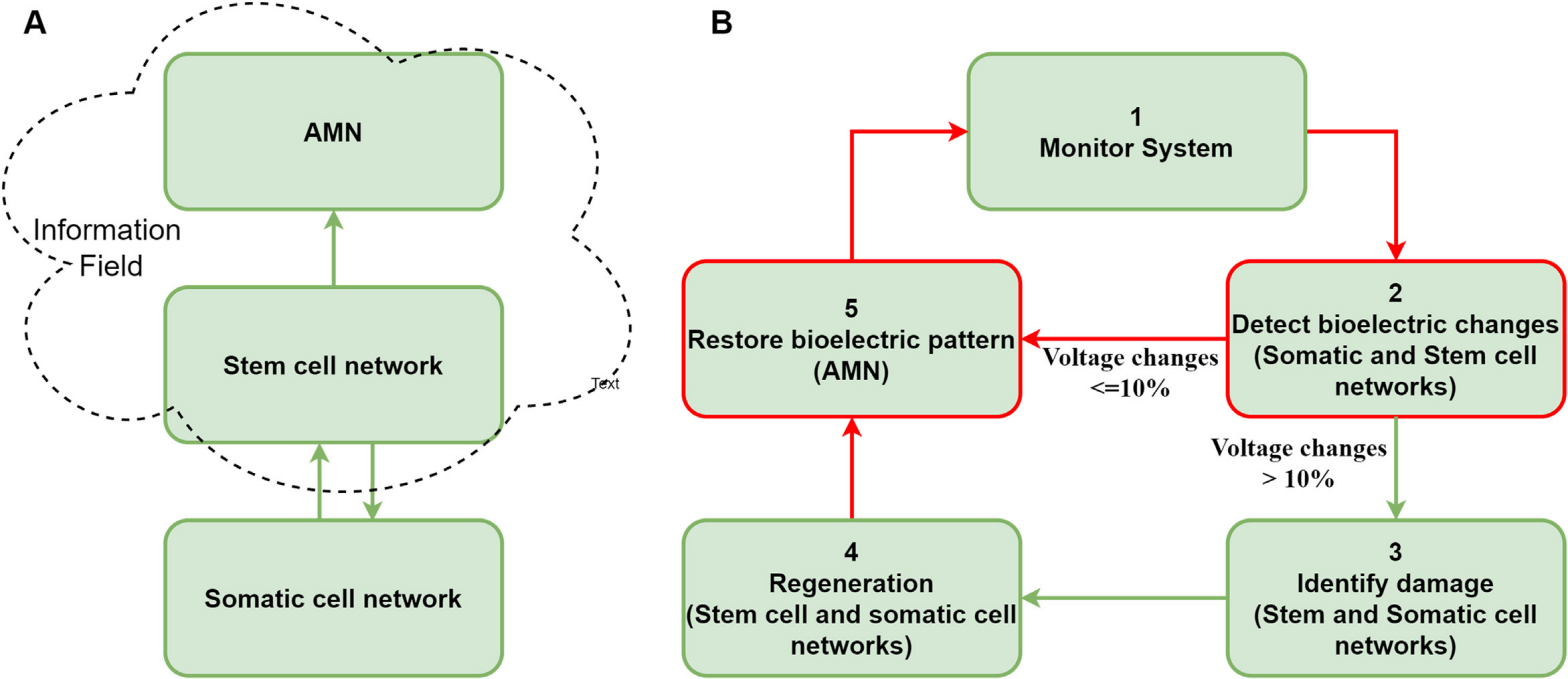
Overview of the framework for anatomical and bioelectric homeostasis. (A) A conceptual basis involving organization of three networks and an Information Field. (B) Functional aspects of the framework involving five stages for restoration of both form and function.

We present the framework in two parts: the conceptual basis and functional description. In part one, we elaborate on the framework's conceptual basis and its organization (Fig. 7A). Part 2 provides a functional description of the framework (Fig. 7B), with algorithmic/computational structures for a complete and accurate restoration of body patterns and bioelectric homeostasis.

### Conceptual basis and organizational view of the self-repair soft robotics framework

We introduce three types of cellular networks in our framework that communicate using bioelectricity to restore both pattern and bioelectric homeostasis (Fig. 7A): the somatic cell networks, the stem cell network, and the AMN that operate as an integrated whole. Specifically, the framework proposes a high-level (macro-level) body-wide AMN as a mechanism for maintaining and restoring voltage homeostasis (level 3 in Fig. 2). This is a recurrent neural network, trained to retain the body-wide bioelectric gradient in its attractor state and recover it from normal perturbations and after damage recovery. The AMN's purpose is to: (1) produce a simple model that maintains the body-wide bioelectric pattern; and (2) be a flexible and fault-tolerant system without excessive micromanagement of the bioelectric state (membrane voltage of cells). As there are approximately 3750 cells, we grouped the cells into clusters, each consisting of a group of stem and somatic tissue cells. We used clusters as AMN nodes (Fig. 2E).

For pattern regeneration, the framework employs somatic cell networks (one for each tissue) (Fig. 2C) and a stem cell network distributed throughout the organism (Fig. 2B). These networks are locally interacting perceptron networks. The stem cell network is the driver or engine of regeneration. We design regeneration mechanisms through bioelectric communication between a large number of stem cells and somatic tissue cells (Fig. 2F). Although voltage varies along and across the organism, there are only three patterns of perceptron communication (interior, border, and corner cells) within the somatic cell networks and the stem cell network. The local interactions and three communication motifs greatly minimize the amount of computation. We also include an information field where the stem cells store a minimal body plan (a few basic tissue dimensions) for recovery from extreme damage, where all exterior boundaries are gone or where there is no trace of the original pattern boundary in the damaged tissue. In our simple worm, the field stores the following pattern information for the three tissues: the length of the body tissue (d), the AR (the length to width ratio) of the head, body, and tail (AR = 1, 3, and 1), and the number (n) of corners of the head, body, and tail (n = 3, 4, 3). All stem cells can access the information field. It also contains the AMN attractor, which corresponds to the homeostasis bioelectric pattern. We present the algorithms and the training and operation of these networks in part 2 of the description.

We retain some features of our previous framework (26) that only regenerated the form (not the function), of the organism. While we retain the perceptron configuration of the somatic cell network, the cell states are derived from actual voltages. Communication is now conducted through bioelectricity instead of binary codes [0,1], and perceptron motifs have trained weights instead of fixed weights. The stem cell network, also represented by a perceptron network, is totally new and much larger, with 375 stem cells distributed throughout the body, instead of the three stem cells represented by fixed linear neurons in the previous framework. Stem cell perceptrons have the same three communication motifs as their somatic cell counterparts. The AMN and the restoration of bioelectric homeostasis are novel. The AMN was trained using a large number of perturbed voltage patterns from the original homeostasis pattern to store the original pattern in its attractor. While the information field is similar, it has been expanded to contain the AMN attractor (the homeostatic bioelectric state). The field can now be accessed by all stem cells and the AMN. Due to the presence of a larger number of stem cells and bioelectric communication, the operating system differs from the previous framework: it seamlessly integrates pattern and bioelectric homeostasis.

### Functional overview of the framework—collective intelligent decision making for autonomous form and function regeneration

The three neural networks operate collectively. They detect the size and location of the damage and repair it and subsequently restore the bioelectric state as presented in the high-level functional view of the system (Fig. 7B). The framework's logic is highlighted in five stages that operate in two modes. The first mode involves restoring bioelectric homeostasis under normal physiological fluctuations in an undamaged organism, where the system monitors (stage 1), detects bioelectric changes in the cells (stage 2), and if the change is smaller than 10% (35) indicative of no damage, it restores the bioelectric state using the AMN (stage 5). The second mode involves damage repair followed by the restoration of bioelectric homeostasis. If the voltage change is greater than the 10% threshold indicative of damage, the framework activates the stem cell network and the affected somatic cell network(s) to determine the damage to respective networks (stage 3). In stage 4, the stem cell and somatic cell networks collaborate to repair the damage. In stage 5, the AMN restores the bioelectric pattern. All three of the system's networks sense changes in the bioelectric state that trigger one of these two modes of operation. Below, we elaborate on these modes of operation before describing the algorithms of the regeneration frameworks.

#### Bioelectric restoration under normal physiological function (stages 2, 5, 1)

When there is a perturbation in the bioelectric state, the system's first task is to check if the voltage change is due to variations under normal physiological function or damage (stage 2). In stage 2, the somatic cells that have experienced a change in their voltage send this information to other cells through the GJs and the surrounding environment (ion fluxes). The stem cells receive this information and decide whether the change is due to damage or not using the ±10% voltage threshold. When it is below 10%, the stem cell network informs the AMN. The AMN works autonomously to restore the organisms’ homeostasis bioelectric pattern by iteratively reaching the stored attractor pattern (stage 5).

#### Anatomical pattern and bioelectric restoration under damage (stages 2, 3, 4, 5, 1)

When there is damage (indicated by a ≥10% voltage change), the stem cell network informs the AMN of this (stage 2). The AMN waits while the affected stem cells check the damage (stage 3) by applying the three perceptron motifs and identifying the stem cell damage border. Affected somatic cells check for missing neighbors and identify the tissue damage border. The somatic cells and the stem cells cooperate to recover the whole pattern from any damage (stage 4). After regenerating, the stem cell network reactivates the AMN to restore the repaired cells’ and the organism's voltage (stage 5). The organism returns to normal anatomical and bioelectric homeostasis (stage 1).

### Algorithms of regeneration

The framework consists of three levels, each with its own computational algorithm: the somatic cell network (level 1), the stem cell network (level 2) and the AMN (level 3). Table S1 in the Supplementary Materials presents the three cell networks, their structure, properties, and activities.

### Level 1: somatic cell network (perceptron network with local communication)

The somatic cell network recognizes the intact pattern and damaged state of individual tissues through local bioelectric communication applied to three *c*ommunication motifs (Fig. S1 and Table S1, Column 2). These motifs specify the rules for the identification of neighbors of a somatic cell (inputs and the corresponding output state of perceptrons). Each somatic cell is represented by a perceptron that has two, three, or four neighbors depending on its location in the somatic cell network (interior, border, or corner, respectively). Therefore, a somatic cell receives two, three, or four inputs from its neighbors. This results in three communication motifs in the somatic cell network (Fig. S1 and Eqns. S1 to S3 in Supplementary Materials). Table S1 provides the generic format of these motifs. We call these motifs because they can be applied to all somatic cells (corner, border, and interior), regardless of where they are located in the organism. As voltage varies throughout the body, only the inputs need to be standardized to 0 or 1 before presenting to the corresponding motif. As the voltage is negative throughout the system (Fig. 2A), 1 indicates the presence of a negative voltage and 0 the absence of any voltage. The output of the perceptron motifs is 0 or 1, indicative of the absence of one or more neighbors or the presence of all neighbors.

Somatic cells learn to recognize their neighbors dynamically, through trained perceptrons. For training, each perceptron motif starts with random values for the bias and input weights (Fig. S1). These weights reflect the normalized electrical conductance of the GJs between somatic cells in a real planarian tissue. Actual conductance of the GJs for the whole organism is not currently known. These model conductance values are normalized representations obtained through training. Bias weight stabilizes the outputs and represents any influence not captured by the inputs. Inputs 0/1 represent the presence or absence of somatic cells based on their voltage. The motifs were trained using Hebbian learning until convergence to a stable state, such that each motif can determine whether a neighbor(s) is present or not. The training was quick and successfully completed (see Table S2 for the three motif weights).

### Identification of tissue damage by somatic cell networks

In the damage state, individual somatic cells, which are neighbors of damaged cells, sense the damage through a change in bioelectricity. They identify damage through the corresponding perceptron motif. The set of somatic cells, which recognize the missing neighbors, become the damage border. Thus, the somatic cell network can identify the borders of any damage (Fig. 5A and B shows tissue damage using a black square that includes a missing stem cell). The tissue cells next to the damage receive an increase in voltage (yellow cells surrounding the back square (Fig. 5B)). Applying the motifs (in this case for interior cells), these tissue cells in the somatic cell network identify that their neighbors are missing and become the damage border. The nearest stem cells sense the border somatic cells’ elevated voltage and check if this is due to normal perturbation or damage.

### Level 2: stem cell network—operation of the regeneration engine (perceptron network with local communication)

The stem cell network (Fig. 2B) is represented by a locally interacting perceptron network. While it is similar to the somatic cell network, it is organism-wide and activated by sensed voltage signals, communicated through somatic cells. Further, these stem cell communications are either activation (+) or inhibition (-) signals depending on their position. From posterior to anterior (right to left), these cells inhibit neighbors. From other directions, they activate neighbors (Fig. 2B). This directed activation and inhibition pattern was extracted from the optimum pattern of nodal interactions found for the AMN network. This pattern is described in the next section, along with justification for the discovered pattern. As stem cells are within AMN nodes, stem cells were made to follow the AMN's interaction format.

The stem cell network recognizes its structure (and the damaged state) using the three perceptron motifs, with the same structure and weights as in the somatic cell network. The neighbors of the missing stem cells form the stem cell border. Depending on the received information, they restore just the bioelectric state (no damage) or both damage and the bioelectric state (after damage).

### Identification of stem cell and large-scale tissue damage

The stem cell network is the primary entity in damage detection and restoration. When stem cells sense increased voltage beyond the threshold, they first have to identify if there is stem cell damage, and if so, whether this is large scale global damage involving completely missing head or body tissues (Fig. 5E) or less destructive damage (local damage) involving one or more stem cells and associated tissue damage (Fig. 5A). Basically, the stem cells must identify the extent of damage to its network and determine the type of damage to the organism. To establish if stem cells have been damaged, this network applies the perceptron motifs to the cells that have experienced changes to their voltage status sensed through intermediate tissue cells. If all the affected stem cells report no missing neighbor stem cells, then the damage involves local tissue only, without stem cell damage. In this case, the somatic cell network identifies the border of the damage in the affected tissue(s) using the communication motifs. The nearest stem cell migrates to the damage border and repairs it. This damage does not involve tapping into the information field as relevant pattern information is contained within the remaining tissue.

Although the forms of damage may be many, the stem cell network applies three pattern primitives to detect all types of stem cell damage. These patterns are based on the nature of the stem cell damage border created by neighbors of missing stem cells in the stem cell network. We explain these under two broad categories: local stem cell damage involving one or more stems cells (without whole tissue loss) (Fig. 5A); and large-scale damage involving the loss of whole tissues causing damage to large segments of stem cells (Fig. 5E). These categories are described in Section 1.3 of the Supplementary Materials.

In brief, the basic concept behind pattern primitives is very simple: all possible stem cell damage border patterns can be constructed using three simple pattern primitives (Fig. 5B). These three patterns refer to whether there are stem cells to the left, right and above or below the damage. In local (Fig. 5A) and open border damage, all three pattern primitives apply, as stem cells are found to the left, right, and above/below the damage (shown in blue circles in Fig. 5A). From this, it is recognized that the damage is local. In the case of large-scale tissue damage (Fig. 5E), only one pattern applies to the stem cell border around the damage. From this, it is recognized that the whole tissue(s) is missing. What exact tissue (the head, body, etc.) is missing is identified by the direction of the flow of missing communication, whether it occurs from the head to the tail or vice versa (described in Section S1.3). For example, in the case of the severed head in Fig. 5E, the stem cell damage border in the head (indicated by the orange arrow) makes a simple pattern of one line of stem cells indicating the presence of stem cells to the left of the damage only. Therefore, only one pattern primitive applies which indicates whole tissue loss. Further, these damage border stem cells do not receive communication from the tail side which indicates that the body is missing. Similar logic applies to the severed tail that does not receive communication from the head side (refer to the respective orange arrow for the stem cell damage border in Fig. 5E). In the case of severed body tissue, there are two locations of damage, on the left and right sides. Each location of damage encompasses only one pattern primitive; that is, for the damage on the right hand side of the body, only pattern one applies, as the stem cells are to the left of the damage. For the damage on the right hand side of the body, only pattern two applies, as stem cells are to the right of the damage, indicating whole tissue loss. Missing communication from both the head and tail sides indicates that the missing tissue is the body.

### Level-3: Global level AMN maintains and restores body-wide bioelectric homeostasis

This section proposes a hypothetical concept for dynamically maintaining bioelectric homeostasis in general functioning and restoring it after damage in an organism like the planarian. We ask what minimal and generic approach could allow the remembrance and restoration of the bioelectric pattern represented by the membrane voltage of thousands of cells in the organism. We propose a global AMN, located on top of the stem cell network in our framework (Fig. 2A level 3 and D). We assume that the body-wide bioelectric pattern is fault tolerant in that small-scale anomalies such as a failure in a single cell does not break the system. Equally, minor voltage fluctuations do not significantly alter the homeostasis voltage pattern. Considering the large number of cells in the organism, a more aggregate form of monitoring, at a macro level, that does not involve all the cells in the required computations is justifiable.

**Fig. 8.**
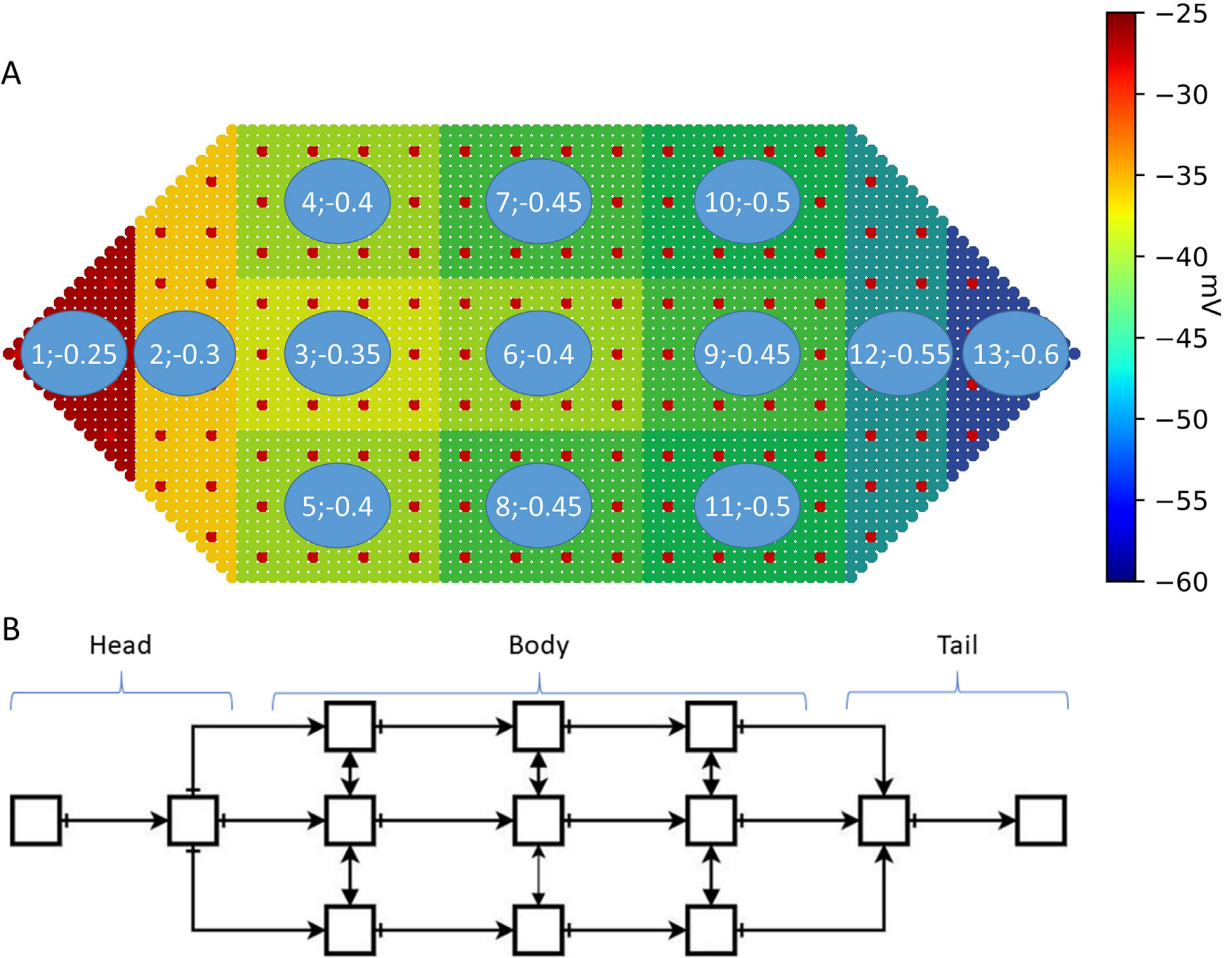
An organism with the original homeostasis bioelectric pattern and the AMN representing it. (A) Innate body-wide bioelectric pattern and 13 tissue segments of the organism used to monitor it. The two numbers in each node are the node number and the corresponding membrane voltage (normalized). Colors indicate the magnitude of voltage (27). The whole voltage pattern across the organism denotes the homeostasis bioelectric pattern; (B) the AMN, consisting of 13 segmented nodes, represents the whole organism and is designed to learn and remember the body-wide bioelectric pattern.

As proof of the concept, we divide the organism into 13 segments, each representing a group of stem and somatic cells with a corresponding level of membrane voltage (Fig. 8A). These constitutes the basic units or nodes of the AMN (Fig. 8B and S3) involved in monitoring and restoring the body-wide bioelectric pattern. In this study, the voltage is assumed to be constant within an AMN node to keep it simple and test the AMN concept. This global-level AMN is a recurrent neural network, which is a modified form of the fully connected Hopfield Neural Network (HNN) (45) that stores specific state vectors in one or a few attractors. We hope to find a network with minimum connectivity (as opposed to the full connectivity of all nodes in HNN) that is sufficient to remember the bioelectric pattern. The benefit of these networks in general is that for any distorted input vector (state vector), the network retrieves the original undistorted form of the same input vector. The AMN operates over the whole anatomical pattern of the organism and learns and memorizes the body-wide bioelectric gradient (Fig. 8A). We provide a summary of the AMN development here (full details are provided in Section 1.4 of the Supplementary Materials).

The voltage pattern shown in Fig. 8A (i.e., 13 voltage values) is the pattern we wish to store in the AMN attractor. When this pattern changes due to normal perturbations or damage, the AMN should recall the original pattern and reestablish bioelectric homeostasis in collaboration with the stem and somatic cell networks. While we do not know if the planarian uses a mechanism similar to the AMN to keep a stable body-wide bioelectric pattern, we do know that the worm maintains a stable pattern. We assume that the approach it uses is flexible and fault tolerant, and that it achieves this aim without excessive micromanagement or extensive memory requirements.

Our proposed AMN has the following attributes. Each AMN node represents a cluster of cells: there are 300 tissue cells and 12 stem cells in a node (the head and tail nodes have a slightly different form to these cells). Each node has a constant voltage. The voltage of all the clusters form the body-wide bioelectric pattern. This arrangement allows the AMN nodes to sense voltage changes in their constituent stem and tissue cells in a global or average sense and react accordingly, to restore the bioelectric pattern under normal function or wait for the stem cell network to initiate repair after injury and complete regeneration. Within a node, neighbor somatic cells are connected by GJs with a particular conductance. Stem cells communicate through somatic cell GJs and ion fluxes. An AMN node containing these cells communicate with neighbor AMN nodes via pseudo GJ connections, reflecting the effective or average conductance of the GJs connecting the somatic cells in the neighbor nodes (Fig. 2D). This system means that the AMN correctly resembles the cellular structure and interactions without losing information due to aggregation. It also helps it to accurately preserve the average membrane voltage profile throughout the body. These pseudo-GJ conductances are represented by the AMN weights that are determined by training. Training is performed through Hebbian learning, with a dataset generated by perturbing the original voltage pattern to represent both normal fluctuations and damage conditions.

Full details of the AMN, its training, and the final network configuration and weights (Table S3) are presented in Section 1.4a of the Supplementary Materials. In brief, the model dynamics use asynchronous neuron state updates with a rectilinear function (ReLU) in the AMN nodes (Fig. S2) that calculates the output (voltage) *s*_i_ of *i*^th^ node as follows (Eq. S5)

(S5) \begin{eqnarray*} {s}_i = \theta \left[ {\mathop \sum \limits_j^N {w}_{ij}{s}_j + {b}_i} \right];\ \theta \ \left( a \right) = \left\{ {\begin{array}{@{}*{1}{c}@{}} {1\ if\ a \ge 1\ }\\ { - 1\ if\ a \le - 1}\\ {a\ otherwise} \end{array}} \right.\, \end{eqnarray*}

where ${w}_{ij}$ is the connection weight between node *i* and *j*, $\theta $ is the transfer function (ReLU), and ${b}_i$ is the bias weight of node *i*. To store the desired pattern, we set random values for the connection weights (*w, b*) and adjusted them using the Hebbian learning rule

(S6) \begin{eqnarray*} {w}_{ij} = {w}_{ij}\ + e*\delta ;\ {b}_i = {b}_i\ + e*\delta ;\ e\ = {T}_i\ - {I}_i, \end{eqnarray*}

where $\delta $ is the learning rate, *e* is the difference between the target pattern *T* and the current input pattern *I*.

The proposed macro-level nodal configuration ensures the monitoring system is fault tolerant, and enables the AMN to represent the whole worm in a smaller representative network with grossly similar bioelectric properties to that of thousands of somatic cells. This configuration greatly simplifies the required computation needed to achieve the end result of maintaining homeostasis, where all somatic cells still contribute to the process. Moreover, by exploring sparse but meaningful connections between nodes in the AMN, we found a much-simplified network configuration that substantially reduces the computational burden on the network. This finding indicates the potential existence of efficient and high-level organism-wide networks for bioelectric control. Studies have shown that bioelectric perturbations can cause large-scale morphological changes, such as the formation of a head in the tail position and a number of other severe anatomical distortions (10, 12).

Basically, with AMN, we do not eliminate anything but instead simplify the system. Since individual somatic cells are not nodes in the network, the network is flexible and fault tolerant. For example, the AMN will only be damaged if all cells in a node are damaged. Until then, the AMN collaborates with the stem cell network within the affected nodes to quickly restore bioelectric homeostasis after repairing any damage to cells within that node. When the perturbation is not due to damage, the network automatically restores the original bioelectric pattern. When a network node is damaged, the network's spatial topology is broken. The network will lose the voltage value of the lost node and the corresponding connection weights. After regeneration, the connections from this node to others need to be reestablished to restore the lost connection weights. After the nodes are repaired, the AMN retrains itself, by accessing the bioelectric pattern from the field to create perturbed input training patterns until the network learns to store the original pattern in the attractor and the connections and weights to the repaired nodes are reestablished.

## Supplementary Material

pgac308_Supplemental_File

## Data Availability

The algorithms and data used in this research have been provided in the Supplementary Material associated with this paper.
