# Supplementary material for: A Comprehensive conceptual and computational dynamics framework for autonomous regeneration of form and function in biological organisms
Source: PNAS Nexus. 2023 Jan 9;2(2):pgac308. doi: 10.1093/pnasnexus/pgac308 (PMC9944231; doi:10.1093/pnasnexus/pgac308)
Supplement: pgac308_Supplemental_File [file pgac308_supplemental_file.docx]

**Supplementary Materials**

**A Comprehensive Conceptual and Computational Dynamics Framework for Autonomous Regeneration Form and Function in Biological Organisms**

**Sandhya Samarasinghe and Tran Nguyen Minh-Thai**

**S1. Further details of the conceptual framework and methods**

**S1.1 Overall view of the components of the new framework and their attributes and properties**

**Table S1. The three cell networks in the framework and their structure, properties and activities**

|  | **Somatic Cell Network** | **Stem Cell Network** | **AMN** |
| --- | --- | --- | --- |
|  | 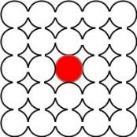 | 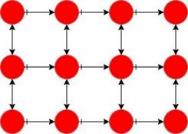 | 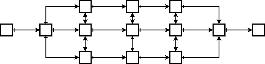 |
| **Network Type** | Perceptron network with local communication | Perceptron network with local communication | Auto-Associative (Associative Memory) network with local communication |
| **Form of Communication** | Somatic – somatic cell (bioelectric, directly through GJs) | Stem cell – stem cell  (bioelectric, indirectly through somatic cell GJs and ion fluxes released to the environment.  (i.e., stem cell *i* ->fluxes-> GJs -> fluxes -> stem cell *j*)  Stem – somatic cell (bioelectric, through somatic cell GJs and ion fluxes released to the environment  (i.e., somatic cell *i* -> GJs -> fluxes -> stem cell *j*) | Node – Node (directly through node connections |
|  | 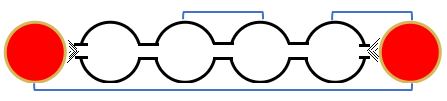 | | 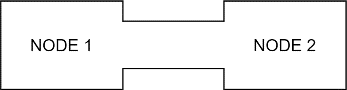 |
| **Output**  **Input** | Cell Status (damaged or not) | | Node Voltage |
|  | Cell Voltage | | Node voltage |
| **Properties** | Three neighbourhood rules for cell location (interior – 4 neighbours, border – 3 neighbours, corner – 2 neighbours)  Accordingly, three perceptron communication Motifs (for 2, 3, or 4 neighbours) trained to represent the three neighbourhood rules for identifying the presence or absence of neighbours (i.e., damage). Solid circles in the figure below represent one motif and the other two are obtained by adding dashed circles one by one. w_i_ represent weights of the motifs.  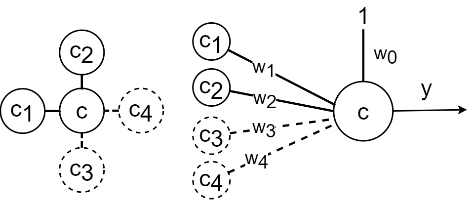 | | - Remembers the whole Bioelectric pattern  -Communication through Network Weights |
| **Information field** | Minimum tissue pattern information (d, AR, n); d is the length of the body tissue; AR is aspect ratio (length/width) of the head, body tail tissues (1,3,1) and n is the number of corners of the three tissues (3,4,3). | | Bioelectric pattern stored in the attractor (normalised):  [-0.25, -0.3, -0.35, -0.4, -0.4, -0.4, -0.45, -0.45, -0.45, -0.5, -0.5, -0.55, -0.6] |

**S1.2 Somatic cell network, communications and repair of local tissue damage**

Somatic cells in each tissue form a somatic cell network. There are head, body and tail somatic cell networks consisting of perceptron neurons that communicate locally. For simplicity of computation, three formats or motifs are defined for this communication as shown in Fig.S1.


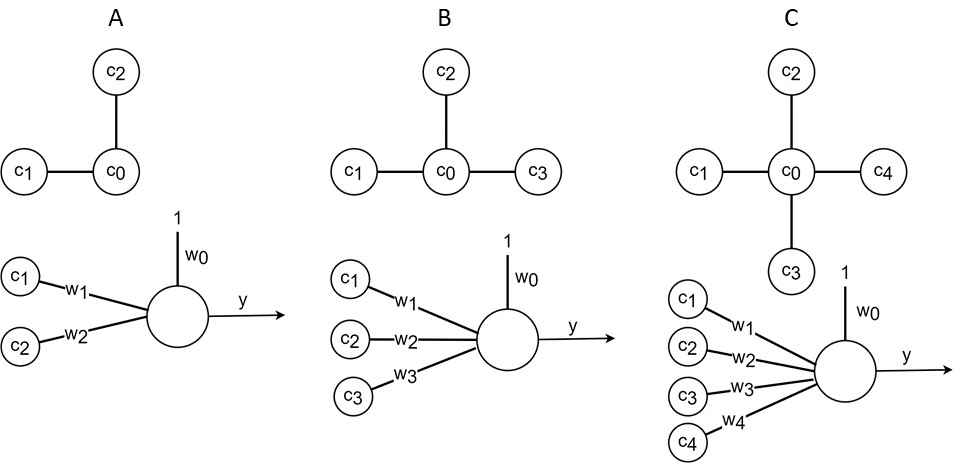


**Fig. S1 Perceptron motifs. Three communication motifs (A, B and C) depending on the number of neighbours 2, 3 or 4, respectively, in the somatic cell network. These denote corner (A), border (B) and interior (C) somatic cells. w_0_ denotes bias weight and w_i_ denotes weights associated with inputs.**

**The three perceptron communication motifs are:**

**Motif 1**: *Rules for a Corner Cell*: Two inputs [c_1_, c_2_] are normal indicative of no damage when [c_1_, c_2_] are non-zero negative (hyperpolarised) voltage values, and other input combinations indicate damage.

$$\begin{aligned} y=\left\{ \begin{aligned} 1 if c_{i}<0; i=1, 2 \\ 0 otherwise \end{aligned} \right.\#\left( S1 \right) \end{aligned}$$

**Motif 2**: *Rules for a Border Cell:*  Three inputs [c_1_, c_2_, c_3_] are normal when [c_1_, c_2_, c_3_] are non-zero negative voltage values, and other input combinations indicate damage.

$$\begin{aligned} y=\left\{ \begin{aligned} 1 if c_{i}<0; i=1, 2, 3 \\ 0 otherwise \end{aligned} \right.\#\left( S2 \right) \end{aligned}$$

**Motif 3**: *Rules for an Interior Cell:* Four inputs [c_1_, c_2_, c_3_, c_4_] are normal when [c_1_, c_2_, c_3_, c_4_] are non-zero negative voltage values, and other input combinations indicate damage.

$$\begin{aligned} y=\left\{ \begin{aligned} 1 if c_{i}<0; i=1, 2, 3, 4 \\ 0 otherwise \end{aligned} \right.\#\left( S3 \right) \end{aligned}$$

When there is local tissue damage with only missing somatic cells, the neighbours of the missing cells experience increased membrane voltage that triggers them identify the missing neighbours by applying the communication motif relevant to them (interior, corner or border cells). These cells with increased voltage become border of the damage. The stem cell(s) nearest to the damage also receive bioelectric communication from the affected somatic cells. This activates the stem cell network and upon finding that there are no missing stem cells, the nearest stem cell migrates to the damage border in the affected tissue and produces new somatic cells to restore the anatomy.

**S1.3 Stem cell network, communication and repair of local and large scale damage**

Stem cell network is an organism-wide network of stem cells represented by perceptrons with local communications. The same three communication motifs of the somatic cell networks apply to this network as well.

With respect to local stem cell damage, refer again to the example damage shown by the black square in Fig. 5A in the main text depicting one missing stem cell (and the surrounding tissue fragment). In this type of damage involving one (or more) stem cell, there still remain stem cells to the left, right, above and below the damage, indicative of local (in this case encased) stem cell damage. To identify all damage scenarios, we identify three generic patterns (primitives) that define the stem cell border of a damage as shown in Fig. 5B. The three primitives specifically refer to if there are stem cells: to the left, right or (above or below) the damage, respectively. Any encased damage would need all three generic pattern primitives and thus presence of all three primitives define the nature of all such local damages without whole tissue loss. For example, in the one stem cell damage case in Fig. 5A, all three patterns apply, as there are stem cells to the left, right and (above and below) the damage, indicating local (i.e., not whole tissue) damage. For other and large scale damages, not all three but one or two pattern primitives define the stem cell border. For example, Fig.5E shows stem cell border patterns (orange arrows) for whole tissue damages. Such damage is identified by the absence of one or more of the above three primitives in defining the stem cell damage border. The identification of damage patterns are specified below.

- - - - - ***Identification of local stem cell damage through primitives applied to the pattern of stem cell border (Figure 5B): All three primitives below should apply***

Pattern 1: stem cell damage border has one (or more) stem cells to the left of the damage in AP (along the body axis) direction.

Pattern 2: stem cell damage border has stem cells above or below the damage (in DV direction).

Pattern 3: stem cell border has one (or more) stem cells to the right of the damage (in AP direction).

- - - - - ***Identification of whole tissue damage through primitives applied to the pattern of stem cell border (Fig.5E): Only one of the above primitives should apply, not all three.***

When not all three pattern primitives described above are present in defining a damage border, it indicates whole tissue damage as in Fig.5E. For example, in the case of severed head tissue in Figure 5E, the stem cell damage border pattern has all stem cells on one side (left for the severed head tissue and right for the severed tail tissue) of the damage; therefore, only one primitive applies and 2 are absent indicating whole tissue damage.

We describe in some detail how these rules apply to identify local stem cell damage in the next section. Then we explain identification of large-scale stem cell damage involving loss of tissues in the following section.

**Identification of local stem cell damage by the stem cell network**

When the stem cell damage is local, the stem cell network finds the border of stem cell damage by applying perceptron motifs and the nature of local stem cell damage by applying the three pattern primitives described above. Specifically, for the damage case shown in Fig. 5A, the neighbours of the missing stem cell in the stem cell network sense a voltage increase beyond the 10% threshold, and by applying perceptron motifs the stem cell network identifies the four neighbours (circled in blue). These are the stem cells that have recognised that their neighbour stem cell is missing from the changes in their voltage status sensed through GJs of the intermediate tissue cells. These then form the border of stem cell damage. Then the above pattern primitives are applied to the damage border and it is evident that all three rules apply as there are border stem cells to the left, above and below, and right of the damage. This indicates local stem cell damage inside the stem cell network. Then the stem cell network regenerates a new stem cell to replace the missing one. Then, repair of damage to the tissue is accomplished in collaboration with the somatic cell network of the affected tissues that identify the damage border in the tissue by applying the perceptron motifs. The somatic cell damage border in the example case of local stem cell damage in Fig.5B is indicated by the yellow somatic cell border surrounding the damaged region (black square).

**Identification of large-scale stem cell damage involving loss of whole tissues by the stem cell network**

When the damage involves whole tissues, the stem cell network identifies the damage border and recognises that there is only one border pattern - all stem cells are on either side of the damage (Fig. 5E). Specifically, the three border pattern primitives are applied to the damage with the result that not all three are valid for this damage. From this, the stem cell networks recognises that a whole tissue(s) is missing. Now the question is how to identify which tissue is missing, head, body or tail, upon which stem cell network regenerates missing tissues while tapping into the information field for the minimum pattern information of lost tissues.

For identification of the type of missing tissues, we define few simple rules based on the communication between stem cells in the stem cell network with activation (positive) and inhibition (negative) signals as shown in Fig.2B. In this network, signals from tail to head (A/P direction) are negative (inhibitory) and in all other directions signals are positive (activation). We use these two features to identify the type of missing tissue(s) as follows:

**Two rules of communication flow for identifying the type of missing whole tissues:**

- Stem cells in the damage border receive **positive signals** but **no negative signals**. This means there is no communication in the tail to head direction (left to right or P/A direction). Therefore,
  - If the border stem cells are in the head, then body is missing.
  - If the border stem cells are in the body, then tail is missing.
- Stem cells in the border receive **negative signals** but **no positive signals**. This means there is no communication from head to tail (right to left or A/P direction)
  - If the stem cells are in the body, then head is missing.
  - If the stem cells are in the tail, then body is missing.

**Examples of application of the three stem cell border pattern primitives and two rules of communication flow for identifying global damages by the stem cell network**

Below we illustrate in summary form how the stem cell network applies the three generic stem cell border pattern primitives and the above two rules of communication flow for identification of the type of missing whole tissues to recognise global damages.

**Example 1**: *Whole tissue loss (body and tail missing)*: The head tissue separates after damage as shown in Fig. 5E (left) leaving body and tail intact. First, the stem cell network identifies the border of stem cell damage in the head tissue, which contains all stem cells on the head side. The network recognises that, of the three pattern primitives, only one applies. This indicates large scale tissue damage. Then, it identifies the type of missing tissue by applying the above rules of communication flow. As these border stem cells are in the head tissue, they **receive positive signals and no negative signals.** From this, the network identifies that the **body tissue is missing**. The stem cell network remaining in the head tissue regenerates body and tail by tapping into the information field for minimum tissue pattern information, upon which they produces body and tail stem cells which produce respective somatic cells to re-establish the stem cell network and somatic cell networks. Similarly, the body-tail part regenerates the head tissue and produces another worm.

**Example 2**: *Whole tissue loss (head and tail missing)*: Let’s consider the body tissue remaining after damage in Fig.5E (middle) leaving three fragments. There are two damage regions: head side and tail side. For each region, the stem cell networks identifies the damaged stem cell border and recognises that there is only one stem cell pattern on either side. As not all three pattern primitives apply, the damage is due to tissue loss. Then, by applying the rules of communication flow to the stem cell border pattern in each damage region, the type of missing tissues are identified. For example, for the severed body tissue, the network identifies that one stem cell pattern **receives negative signals but not positive ones** (from head side) and vice versa for the tail side. Thus it identifies **head missing on the left side and tail missing on right side of damage**. The border stem cells then extracts the minimal pattern information for head and tail from the information field and the stem cells on the head side produce new stem cells which produce new somatic cells to regenerate the head using the minimal pattern information. Similarly, the stem cell network on the tail side produces new stem cells that regenerate the tail to the required pattern. Similar logic applies to the regeneration of the severed head and tail tissues. Single stem cell border pattern in these tissues defines whole tissue damage and absence of signals from the body side for the head fragment indicates missing body and tail and vice versa for the tail fragment. In both these cases, the respective border stem cells extract the minimal tissue plans from the information field and regenerate new stem cells to produce new somatic cells to match the pattern requirements for the missing tissues. Thus stem cell network fragments remaining after damage regenerate three worms from the three split segments.

**S1.4 Associative Memory Network (AMN)**

**a. AMN training**

The original HNN (Hopfield Neural Network) is a fully connected and symmetric (symmetric means weights for forward and backward interactions between two nodes are the same) recurrent network with two discrete values for states ${(s}_{i}=\pm1).$ When trained with Hebbian learning, these networks converge to at least one attractor. Our voltage input pattern vectors, however, are not discrete values of $\pm1$but a number of real (continuous) values. In [28], the authors used continuous state HNN with $s_{i}[-1,+1]$ and a fully connected asymmetric connection matrix. This continuous-state asymmetric network also trained well with Hebbian learning and converged into attractors from any inputs. Therefore, in our model, we use real-valued inputs, and we further explore beneficial and potentially meaningful connection configurations as an alternative to fully connected networks to reduce the computational burden of the network in having to work with a large number of weights.

Inputs in our model are real voltage values corresponding to the states of the 13 nodes in the network and scaled to $s_{i}[-1, 0]$ (minus values represent a hyperpolarised (more negative potential inside the cell than outside) voltage state of the cell membrane). Fig.8A (in the text) shows the original homeostasis bioelectric pattern in planaria represented by the 13 AMN nodes. The desired pattern of bioelectric gradient is in the range from -25mV to -60mV (milli Volts) which has been reported as the pattern of bioelectric homeostasis in planaria [29, 30]. In our model, the desired homeostasis bioelectric pattern for the organism consists of the 13 scaled voltage values of (-0.25, -0.3, -0.35, -0.4, -0.4, -0.4, -0.45, -0.45, -0.45, -0.5, -0.5, -0.55, -0.6) shown in Fig.8A. These correspond to the voltage values of the 13 macro-level nodes and represent the desired network attractor. We trained the AMN with a large number of voltage patterns (approximate 600) randomly perturbed from the original voltage pattern in Fig.8A. On a side note, these perturbed voltage patterns can correspond to voltage patterns that can result from the perturbation of parameters in a set of Differential Equations (Eq. S4) representing bioelectric communication between somatic cells via gap junctions as in [31].

$$\begin{aligned} C_{i}\frac{{dV}_{i}}{dt}={-I}_{in}\left( V_{i} \right){-I}_{out}\left( V_{i} \right)+\sum_{j nn i} G_{ij}\left( V_{j}-V_{i} \right)+\sum_{j nn i} C_{ij}\left( \frac{{dV}_{j}}{dt}-\frac{{dV}_{i}}{dt} \right)\#S4 \end{aligned}$$

where $V_{i}$is the membrane voltage of cell *i*, $G_{ij}$ is the conductance of GJ between cells i and j ($C_{ij}$ is capacitance), nn is the number of nearest neighbours of cell *i*, $I_{in (or out)}\left( V_{i} \right)$ is the current-voltage curve, and $C_{i}$is a constant; for details of these attributes refer to [31].

We simply highlight this ODE model to point out that the AMN in functionality (not in structure) represents existing bioelectric models of cell networks on a large scale (organism-wide). In future, this connection between the ANN and ODE models can be exploited to integrate cellular bioelectric and even molecular models into the framework (i.e., not only bioelectric models but also models of slow diffusion of molecules carrying signals for cell division in regeneration).

In training, the AMN starts with random connection weights that allow for learning the desired bioelectric pattern. Basically, we wish the AMN to store the target homeostasis bioelectric pattern in its attractor and invoke this pattern in response to any (altered) input voltage pattern vector. Therefore, all input patterns generated by perturbing the original voltage pattern should form the basin of attraction of the attractor. The learning algorithm goes through the following steps: (1) assignment of the initial states to the neurons (from the generated input patterns), (2) convergence of the network for a certain period (network simulation) followed by (3) application of Hebbian learning that adjusts weights incrementally until the network learns (stores) the target voltage pattern in an attractor and correctly recalls it from any input voltage condition that the organism may encounter.

The model dynamics use asynchronous neuron state updates with a rectilinear function (ReLU) in the nodes (Fig.S2) that calculates the output (voltage) *s*_i_ of *i*^th^ node as follows (Eq. S5):

$$\begin{aligned} s_{i}=\theta\left[ \sum_{j}^{N} w_{ij}s_{j}+b_{i} \right]; \theta\left( a \right)=\left\{ \begin{aligned} 1 if a\geq1 \\ -1 if a\leq-1 \\ a otherwise \end{aligned} \right.\#\left( S5 \right) \end{aligned}$$

where $w_{ij}$ is the connection weight between node *i* and *j,* $\theta$ is the transfer function (ReLU) and $b_{i}$ is the bias weight of node *i*. In order to be able to store the desired pattern, we set random values for the connection weights (*w, b*) and adjusted them using Hebbian learning rule:

$$\begin{aligned} w_{ij}=w_{ij}+e*\delta; b_{i}=b_{i}+e*\delta; e=T_{i}-I_{i}\#\left( S6 \right) \end{aligned}$$

where $\delta$ is the learning rate, *e* is the difference between target pattern *T* and current input pattern *I*. We used a learning rate of 0.1 to make sure that solution space is traversed comprehensively during training.

**Fig.S2. Rectilinear function for output (voltage) s_i_ of a node in AMN**

We designed the structure of the network by iteration. We first used a fully connected network as in HNN where all nodes communicate with each other. As this requires a massive amount of calculation to find an attractor with a large number of connection weights, we then reduced the connectivity in a number of stages to explore a simpler yet meaningful connection configuration that stores and recalls the pattern with minimum computation. We found a minimal connection configuration where local communication prevails as in Fig.S3 and where both the computation and memory storage requirements are drastically reduced. This resulted in 40 connection weights, a reduction of 87% from 312 connection weights in the fully connected network. This means that the reduced network has only 12% of the connections of the full network. The trained weights of the AMN are given in Table S3.

The minimal network topology that remembers and recalls the original homeostasis bioelectric pattern is as follows:

- Connections from head to tail and in the transverse (from the midline to edges) directions are positive (activation signals).
- Connections from tail to head are negative (inhibitory signals).


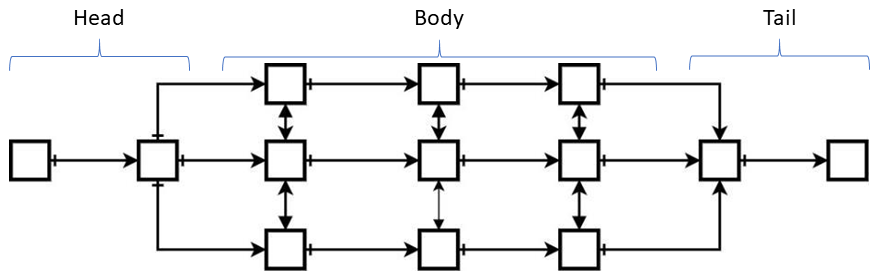


**Fig. S3. A minimal AMN structure that stores and recalls the original bioelectric pattern. Activation signals from head to tail (A/P) and transverse directions and inhibitory signals from tail to head (P/A) direction**

The fact that such a simplified and organised structure emerged was intriguing. Specifically, this communication structure means that a node only receives signals from adjacent cells as follows:

- **For Anterior-posterior (AP) axis (longitudinal direction of the body):**
- Positive signals: flow from head to tail
- Negative signals: flow from tail to head
- **For the Dorsal -ventral (DV) axis (transverse direction of the body):**
- Positive signals flow in both directions from mid-body to edge

Recall that this communication structure between nodes was adopted for the stem cell network as well as described in the text. Further, the above signal flow structure along and across the whole organism was used to derive the rules for identification of the type of missing whole tissues by the stem cell network as presented in the previous section.

**b. AMN maintains bioelectric homeostasis under regular perturbations**

In this section, we first discuss details of the algorithms and computations involved in the restoration of bioelectric homeostasis under normal physiological conditions and then present those involved in its restoration after recovery from damage.

The voltage of each AMN node $\boldsymbol{V}_{\boldsymbol{k}}$ is calculated by the following formula:

$$\begin{aligned} \boldsymbol{V}_{\boldsymbol{k}}\boldsymbol{=}\frac{\boldsymbol{1}}{\boldsymbol{n}}\sum_{\boldsymbol{i=1}}^{\boldsymbol{n}} \boldsymbol{v}_{\boldsymbol{i}}\boldsymbol{\#}\left( 7 \right) \end{aligned}$$

where n is the number cells in a node (e.g., n=300 cells), $\boldsymbol{v}_{\boldsymbol{i}}$ is the voltage of cell *i*, and *k* is the node number $k [1,13]$. Fig. 3A (and 8A) in the text shows the unperturbed original bioelectric pattern. Fig.3B shows an example of perturbed voltage in a single node where one or few of its cells experience voltage change, and Fig.3C shows the perturbed voltage in all nodes where all cells experience voltage changes.

**b (i) A single cell in a node receives perturbation to its voltage**

Cells may be affected by their surroundings, such as temperature, light, chemicals and processes inside the cells, during normal physiological function, causing the voltage to change. According to the literature, membrane voltage can change up to ± 10% during normal operation [26]. We assume a threshold of ±10% for normal perturbations of membrane voltage of a cell.

Let $\boldsymbol{\epsilon}$ be the voltage change in a single cell. The updated voltage in node k is presented as (e.g., Fig.3B):

$$\begin{aligned} V_{k}=\pm\epsilon+\frac{1}{n}\sum_{i=1}^{n} v_{i}; \epsilon=\frac{vp}{n}\#\left( S8 \right) \end{aligned}$$

where, $\boldsymbol{p}$ is the percentage of voltage change, e.g. -10% to 10%, and v is the original voltage of a single cell.

**b (ii) Many cells receive voltage perturbations**

In the case where many cells undergo change in voltage (e.g., Fig.3C), the total voltage change in a node N_k_ (k=1, 2, .., 13) is computed as follows:

$$\begin{aligned} \epsilon=\sum_{i=1}^{j} \frac{v_{i}p}{n} \#\left( S9 \right) \end{aligned}$$

where j is the number of cells in node N_k_ receiving voltage perturbations

The algorithm for AMN recovery of the original voltage pattern from perturbed voltage vector is as follows:

- Determine the current altered voltage vector for the nodes (using Eq. S8 or S9).
- Present the current voltage pattern as input to AMN
- AMN iterates a number of times until it reaches a steady state which is the original bioelectric pattern stored in the attractor.
- In normal physiological functioning, the network is assumed to make affected nodes (specifically, affected cells within nodes) incrementally alter the bioelectric state until they return to the normal state. In the real organism, this could be achieved by the bioelectric activation of the required molecular signalling networks that manipulate membrane bound ion channels to adjust the cell voltage.

**c. AMN maintains bioelectric homeostasis after regeneration**

The AMN also restores the bioelectric pattern after the stem cell network completes regeneration. Recall that an AMN node consists of 300 somatic cells and 12 stem cells. In case of small damages where any single AMN node is not completely damaged (i.e., not all cells within a node get damaged), the AMN can restore bioelectric state after regeneration of missing somatic or stem cells in individual nodes. However, when an AMN network node gets damaged, the spatial topology of the network is broken. The network will then lose the node and all connection weights to this node. After completing regeneration, the connections from this node to others need to be re-established to restore lost connection weights. Further, the voltage of repaired nodes will not be the same as the required and therefore, nodal voltage also needs to be updated. We assume that after the initial training of the AMN, the original bioelectric pattern (bioelectric gradient) captured by the attractor is stored in the Information Field along with the connection topology as a template. After nodes are repaired following injury, the AMN (Fig.S3) retrains itself by accessing the bioelectric pattern from the field until the network learns to store it in the attractor and the connections and weights to repaired nodes are re-established. The algorithm is as follows:

- Initialise connections: Connections from the new node to others are re-established based on the network topology, and corresponding weights are initialised with random values (e.g., 0.1 for positive, -0.1 for negative signals).
- Retrain the AMN: The network retrains with the target bioelectric pattern accessed from the Information Field. Here the original pattern is perturbed to produce training patterns. (since the network has to train only the damaged connection weights, it is trained faster than the original network)
- Restore weights to repaired nodes.
- Adjust voltage: Cells in the affected node(s) alter the bioelectric state incrementally until they return to the normal equilibrium state.

In the text, we describe the operation of the whole framework integrating the three levels.

**S3. Some Results**

**Table S2: Weights of perceptron motifs**

|  | **Bias** | **Input 1** | **Input 2** | **Input 3** | **Input 4** |
| --- | --- | --- | --- | --- | --- |
| **Motif 1** | -0.01 | 0.01 | 0.01 |  |  |
| **Motif 2** | -0.2 | 0.1 | 0.1 | 0.1 |  |
| **Motif 3** | -0.05 | 0.01 | 0.01 | 0.03 | 0.01 |

**Table S3: Weights of Associative Memory Network (AMN) (top part shows weights for connections between nodes and the bottom part shows bias weights of nodes)**

| **Node** | **1** | **2** | **3** | **4** | **5** | **6** | **7** | **8** | **9** | **10** | **11** | **12** | **13** |
| --- | --- | --- | --- | --- | --- | --- | --- | --- | --- | --- | --- | --- | --- |
| **1** | 0.0 | 0.01 | 0.0 | 0. 0 | 0.0 | 0.0 | 0.0 | 0.0 | 0.0 | 0.0 | 0.0 | 0.0 | 0.0 |
| **2** | -0.86 | 0.0 | 0.01 | 0.01 | 0.01 | 0.0 | 0.0 | 0.0 | 0.0 | 0.0 | 0.0 | 0.0 | 0.0 |
| **3** | 0.0 | -1.0 | 0.0 | 0.01 | 0.01 | 0.01 | 0.0 | 0.0 | 0.0 | 0.0 | 0.0 | 0.0 | 0.0 |
| **4** | 0.0 | -1.0 | 0.01 | 0.0 | 0.0 | 0.0 | 0.01 | 0.0 | 0.0 | 0.0 | 0.0 | 0.0 | 0.0 |
| **5** | 0.0 | -1.0 | 0.01 | 0.0 | 0.0 | 0.0 | 0.0 | 0.01 | 0.0 | 0.0 | 0.0 | 0.0 | 0.0 |
| **6** | 0.0 | 0.0 | -1.0 | 0.0 | 0.0 | 0.0 | 0.01 | 0.01 | 0.01 | 0.0 | 0.0 | 0.0 | 0.0 |
| **7** | 0.0 | 0.0 | 0.0 | -1.0 | 0.0 | 0.01 | 0.0 | 0.0 | 0.0 | 0.01 | 0.0 | 0.0 | 0.0 |
| **8** | 0.0 | 0.0 | 0.0 | 0.0 | -1.0 | 0.01 | 0.0 | 0.0 | 0.0 | 0.0 | 0.01 | 0.0 | 0.0 |
| **9** | 0.0 | 0.0 | 0.0 | 0.0 | 0.0 | -1.0 | 0.0 | 0.0 | 0.0 | 0.01 | 0.01 | 0.01 | 0.0 |
| **10** | 0.0 | 0.0 | 0.0 | 0.0 | 0.0 | 0.0 | -1.0 | 0.0 | 0.01 | 0.0 | 0.01 | 0.0 | 0.0 |
| **11** | 0.0 | 0.0 | 0.0 | 0.0 | 0.0 | 0.0 | 0.0 | -1.0 | 0.01 | 0.0 | 0.0 | 0.01 | 0.0 |
| **12** | 0.0 | 0.0 | 0.0 | 0.0 | 0.0 | 0.0 | 0.0 | 0.0 | -1.0 | -1.0 | -1.0 | 0.0 | 0.01 |
| **13** | 0.0 | 0.0 | 0.0 | 0.0 | 0.0 | 0.0 | 0.0 | 0.0 | 0.0 | 0.0 | 0.0 | -1.0 | 0.0 |

| **Node** | **1** | **2** | **3** | **4** | **5** | **6** | **7** | **8** | **9** | **10** | **11** | **12** | **13** |
| --- | --- | --- | --- | --- | --- | --- | --- | --- | --- | --- | --- | --- | --- |
| **Bias** | -0.243 | -0.5 | -0.634 | -0.688 | -0.688 | -0.732 | -0.837 | -0.837 | -0.83 | -0.936 | -0.936 | -1.99 | -1.145 |

**S3 Further examples of implementation of the regeneration framework – Recovery from complex damage cases**

This section presents few more examples of small and large local damages and few complex and severe global damage cases and how the framework successfully and completely regenerates the whole worm.

**Case 1: Multiple somatic cells missing**

The case of more than one somatic cell missing is similar to one cell missing (Fig.S4). However, more than one stem cell may check for damage and implement recovery. Fig.S4A illustrates an example case where five somatic cells (black) have been lost. The somatic cell network determines the border of damage (yellow colour cells in Fig. S4B. Here, three nearest stem cells recognise that there constituent somatic cells have been damaged (Fig.S4B burgundy cells) and recognise what/where the damage is. Here, only somatic cells are gone, and each stem cell repairs the tissue in its neighbourhood (Fig.S4C), and then AMN restores the original voltages (Fig.S4D).


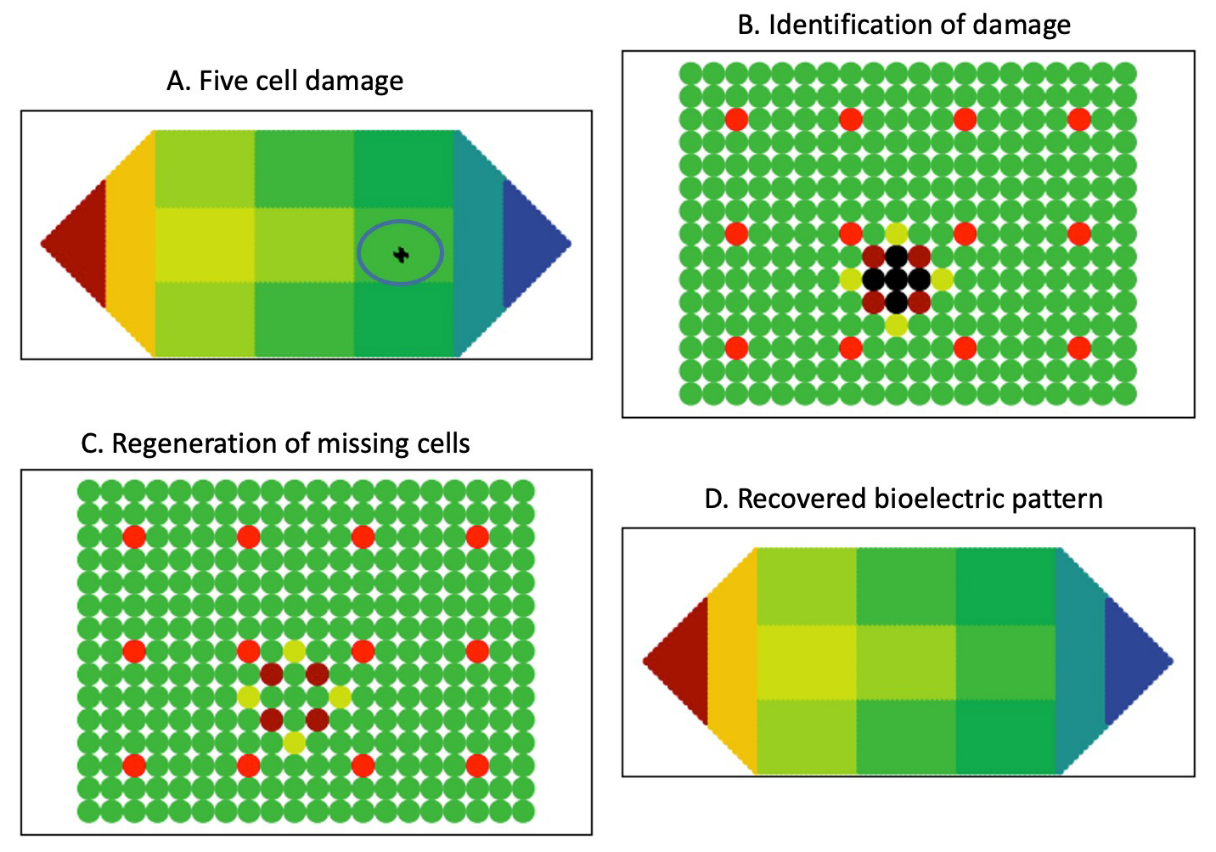


**Fig. S4. An example of five somatic cell damage – (A) Five damaged somatic cells (black cells); (B) Damage identification: damaged cells and their neighbours (yellow colour cells; yellow indicates that voltage in the region is higher than normal); burgundy cells and red cells respectively are stem cells in the affected area and unaffected stem cells; (C) Regeneration of missing cells; the voltage of new cells are equal to the voltage of the producing (burgundy) stem cells; (D) AMN recovers the original bioelectric state**

**Case 2: Organism recovers from damage to many stem cells and surrounding tissue – an AMN node damage**

**Damage identification and recovery:**

This case involves a relatively large damage (about 300 cells) involving 12 stem cells and surrounding somatic cells in the interior of the body as shown in Fig.S5A and in the magnified view of damage region (black square) in Fig.S5B. This is damage to a node in the AMN. The methods of sensing, identifying and repairing the damage are similar to the methods for the previously presented case of a stem cell and surrounding tissue damage. Specifically, the stem cell network first identifies missing stem cells and the damage border in the stem cell network (burgundy colour dots (stem cells) surrounding the black square in Fig.S5B). As all three pattern primitives apply, the stem cell damage is recognised as local. The somatic cell network identifies missing neighbour somatic cells and identifies the damage border in the tissue (yellow cells surrounding black square), using the perceptron motifs. Fig.S5C-D show few snapshots of regeneration for this damage case. Specifically, the border stem cells migrate to the damage site and produce 12 new stem cells that generate somatic cells to fill the space left by the damage.

**Restoration of biolectric homeostasis**

As a node in the AMN is gone, we apply the algorithm in section S1.4c above to restore bioelectric homeostasis.


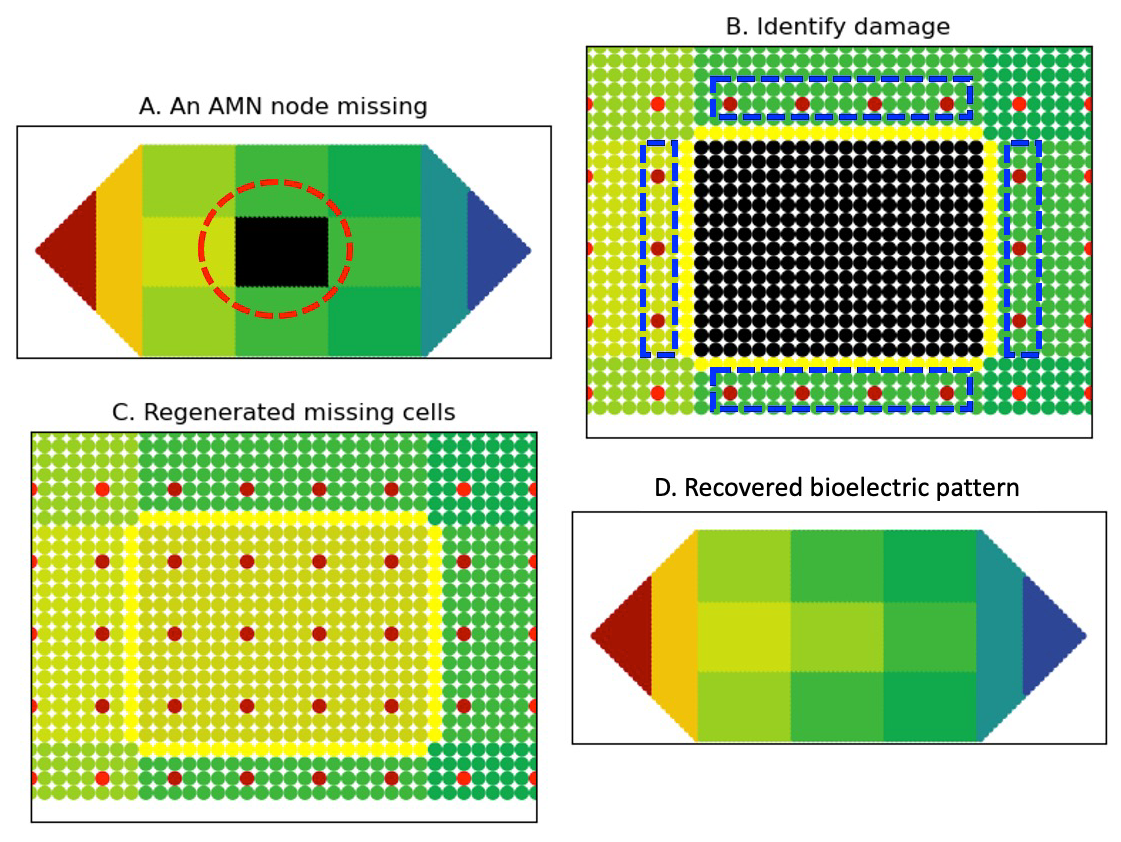


**Fig.S5. Large damage of the size of an AMN node including 12 stem cells and 300 somatic cells. (A) Damage region (black square) where cells are missing; Dash lines highlight the affected cell area; (B) Somatic cell network identifies border surrounding tissue damage (yellow border) and stem cell network identifies its damage border (burgundy dots surrounding the damage); yellow and burgundy indicate that voltage in the region is higher than normal); (C) damage area and surrounding tissue after regeneration (D) AMN restores the body-wide bioelectric pattern.**

**Case 3: Organism recovers from separated whole tissues along with interior damage**

Fig.S6 depicts an injury severing head and tail from the body and further incurring damage to the interior of the body of the worm (Fig.S6A). This is a combination of three damages: head, tail, and a group of interior body cells (AMN node in this case) missing. Figure S6B shows the group of cells removed from the body of the worm.

For the damage in Fig.S6A, the three combined damages mean that it requires a combination of three corresponding recovery processes. Using the three border pattern primitives, stem cells identify the two ‘whole tissue’ damages and the ‘local damage’ to the tissue. The worm achieves head and tail regeneration as in the Case 3 – Head regenerates body and tail shown in the text and missing piece regeneration as in Case 2 (above) for missing AMN node. Fig.S7 shows few snapshots in the repair of the worm starting from the remaining body tissue as in Fig.S6A and the final step of restoration of the voltage pattern.


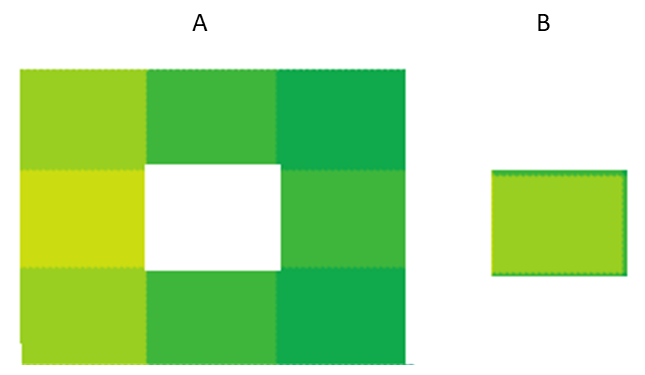


**Fig.S6. Complex damage: A) Separation of parts (head and tail) and a portion of the body interior; B) portion separated from the body interior (A block represents an AMN node)**

Fig.S8 shows the regeneration of the full worm from the small interior part separated from the body as in Fig.S6B. The worm achieves this by applying the same procedures as in Case 3 – Head regenerates body and tail (in the text). Specifically, after determining the border and missing tissues (Stages 2, 3), the stem cells proceed to restore the head and tail (stage 4) (Fig.S8A). Head and tail regenerate concurrently starting from small tissues as a small worm (Figure S8B-D) that grows into the exact original form following shape information taken from the Information field (Fig.S8E). After regeneration, the AMN is re-established, and the bioelectric pattern is restored (Fig.S8F) (stage 5).


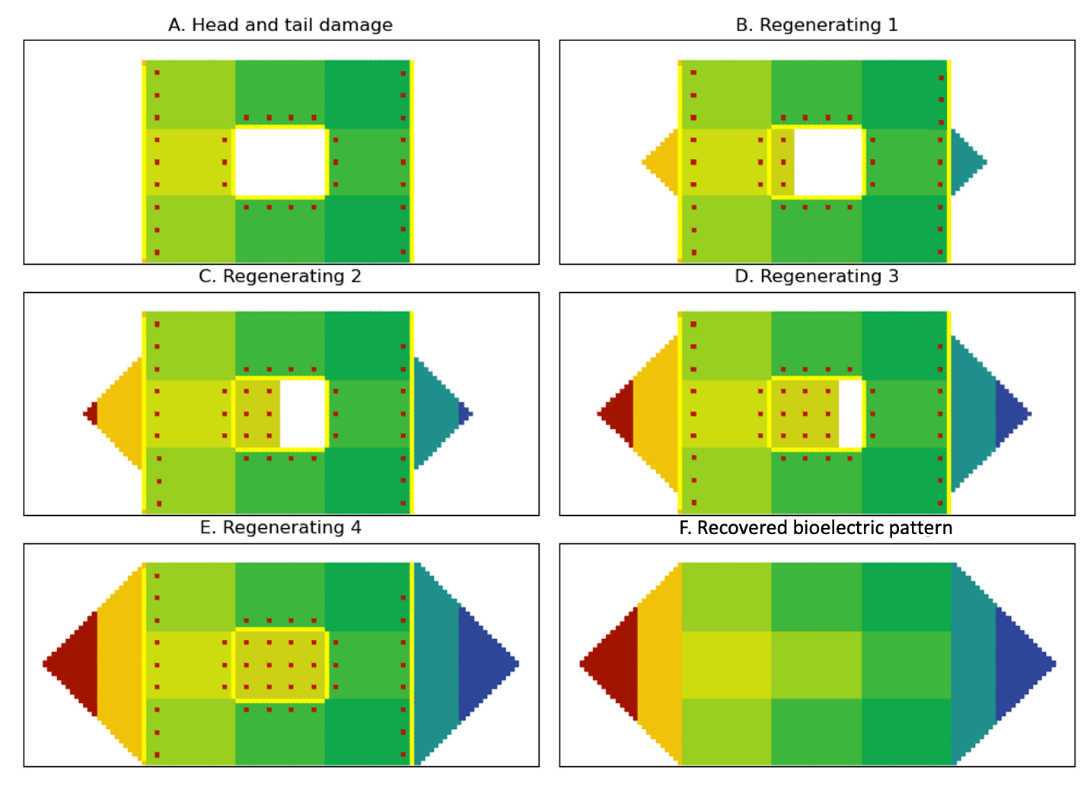


**Fig. S7. Few snapshots from the process of regeneration from damage in Fig.S6A**


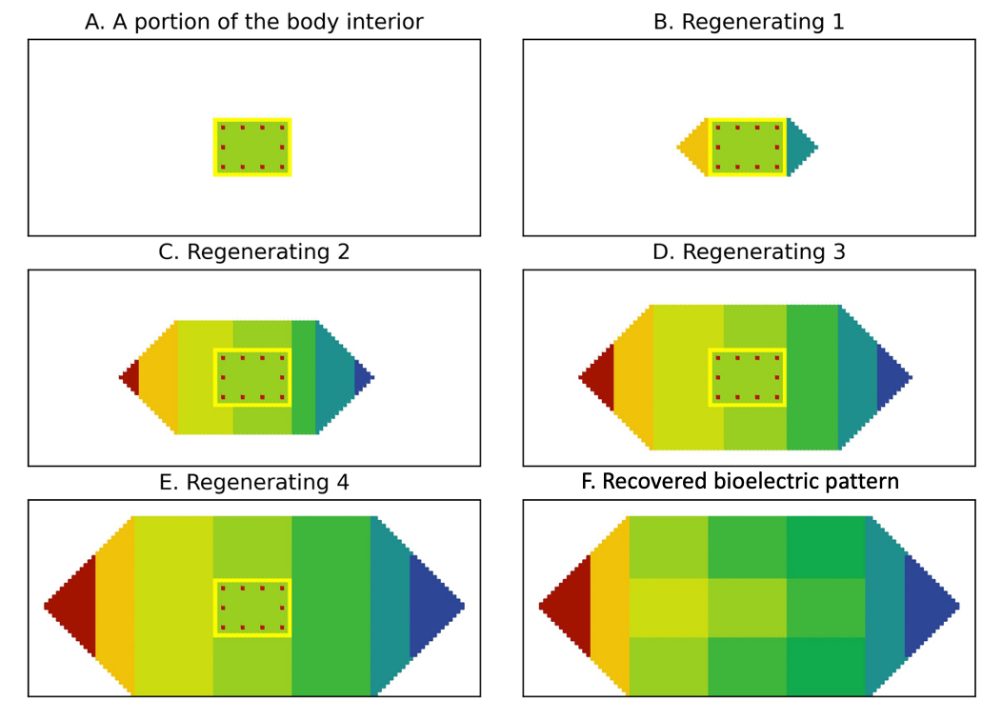


**Fig.S8. Regeneration of the full worm from the small fragmented part in Fig.S6B**

**Case 4: Complex damage boundary**

Fig.S9 presents another complex damage case where the worm is split into two with a vertical cut part-way through the body and then a horizontal cut through the body and tail: one part consists of complete head tissue and parts of body and tail tissues and the other part consists of the rest of the body and tail parts.


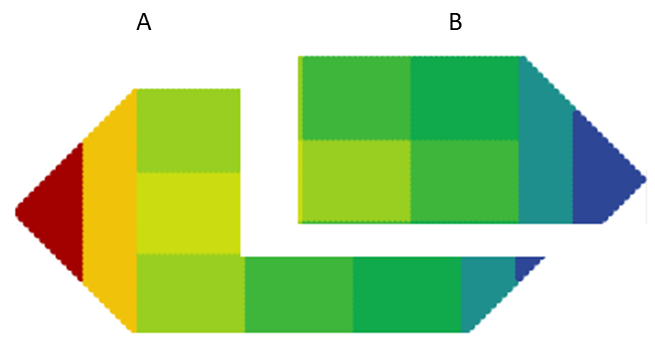


**Fig.S9. Complex damage splitting the worm into two through a number of issues. (A) Part 1: Intact head with body and tail damage. (B) Part 2: No head and body and tail damage.**

Part 1: As shown in Fig.S9A, after damage, there is still the complete head, about half the body, and a portion of the tail tissue left. As such, this is not whole tissue damage. Stem cells identify and repair damage as the case of ‘many stem cells missing’. The shapes of the body and tail tissues are determined from the current borders and length of sides (d) accessed from the Information field. Regeneration steps for this case are shown in Fig. S10.

Part 2: There is still about half the body and a large portion of tail tissue left after damage (Fig.S9B). As such, this is a combination of whole and partial tissue damage. The process of damage detection and identification is as in the above cases. Specifically, stem cells identify the head missing (whole tissue loss) and damage to the body and tail parts (local/partial damage). Stem cells get pattern information for the new head from the information field (length (d), aspect ratio AR, corners (n)) and regenerates a head. The repair of the body and tail relies on the pattern information retained in the damaged tissue and extracting the length and width information from the field. Regeneration steps of this damage are shown in Fig.S11.

**
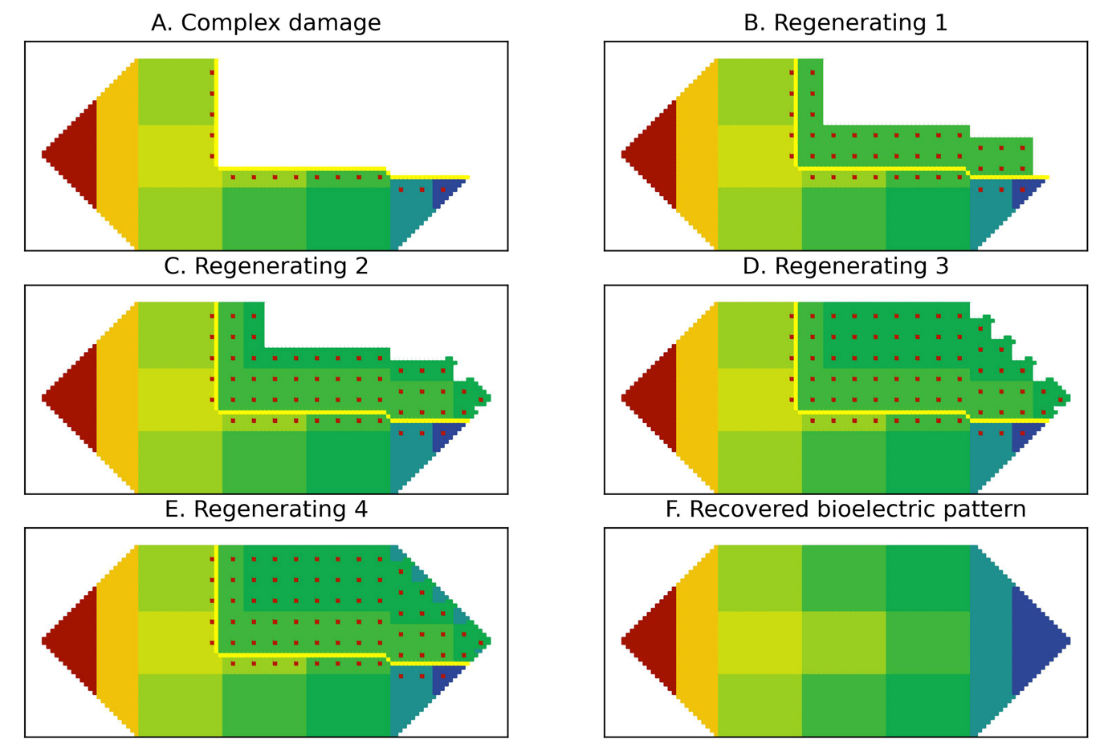
**

**Fig.S10. Regeneration process for damage in Fig.S9A**

**
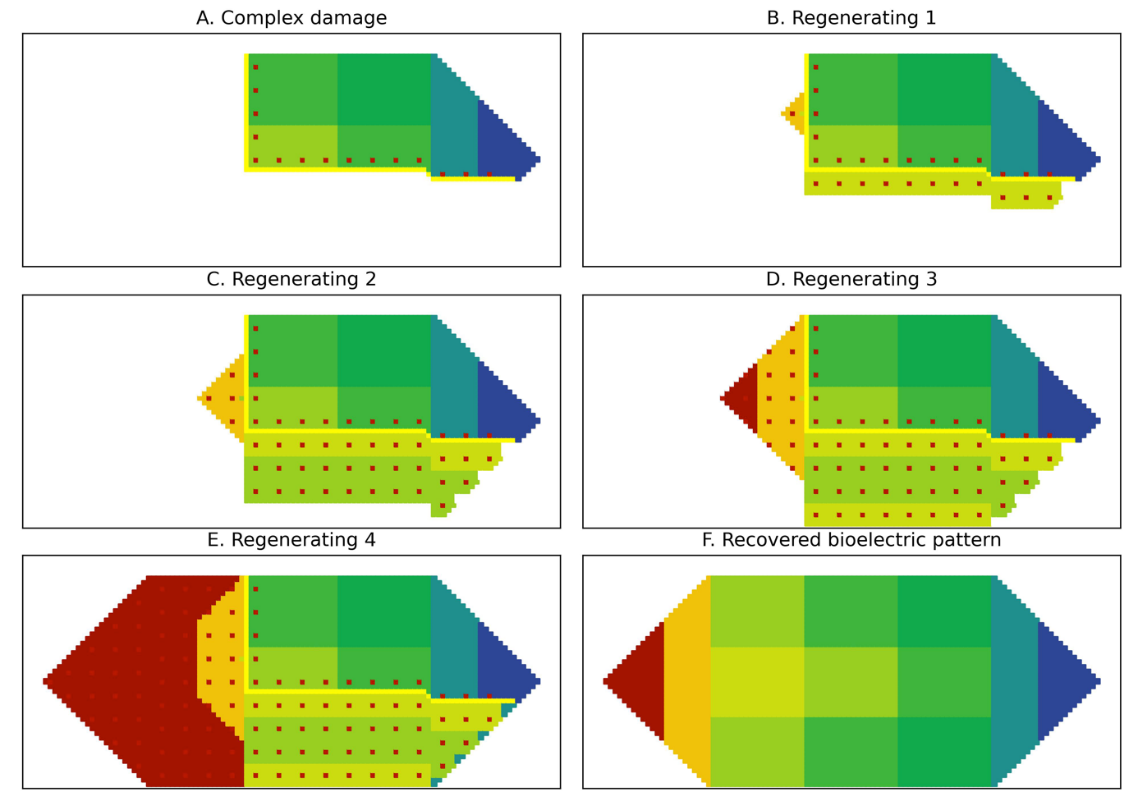
**

**Fig.S11. Regeneration steps for the damage in Fig.S9B**
